# Membranes Based on Metal–Organic Framework Nanostructures for Recovering Nickel, Cobalt, and Manganese Ions from Spent Lithium-Ion Batteries

**DOI:** 10.1021/acsanm.5c04698

**Published:** 2025-12-18

**Authors:** Waseem Iqbal, Amira Nour, Rosaria Bruno, Pietro Magarò, Carmine Maletta, Rosangela Elliani, Antonio Tagarelli, Teresa F. Mastropietro, Jesús Ferrando-Soria, Emilio Pardo, Donatella Armentano

**Affiliations:** † Dipartimento di Chimica e Tecnologie Chimiche, Università della Calabria, 87030 Rende, Cosenza, Italy; ‡ Dipartimento di Ingegneria Meccanica, Energetica e Gestionale, 18950 Università della Calabria, 87030 Rende, Cosenza, Italy; § Instituto de Ciencia Molecular (ICMOL), Universitat de València Paterna, 46980 València, Spain

**Keywords:** metal−organic framework, mixed matrix membranes, metal ion recovery, lithium-ion batteries, MOF-based technologies

## Abstract

Lithium-ion batteries (LIBs) are central to sustainable energy technologies, and while lithium (Li) is the most recognized component, its rapid growth has also intensified demand for other critical metals, particularly manganese (Mn­(II)), nickel (Ni­(II)), and cobalt (Co­(II)). Recovering these valuable metal cations from spent LIBs and associated wastewater is essential to reduce production costs, conserve resources, and mitigate environmental risks. Here, we present mixed matrix membranes (MOF-PES MMMs) as versatile adsorbents for the simultaneous recovery of Ni­(II), Co­(II), and Mn­(II) from multicomponent solutions. Five metal–organic frameworks (MOFs)—ZIF-8, MIL-53­(Al), UiO-66, and their amino-functionalized analogues (NH_2_-MIL-53­(Al) and NH_2_–UiO-66)—were synthesized and incorporated into poly­(ether sulfone) (PES) membranes. The choice of MOF filler significantly influenced metal affinity, with amino functionalization enhancing adsorption relative to the parent structures. Among all formulations, ZIF-8-PES exhibited broad-spectrum performance, achieving >90% removal efficiency for all three metal ions simultaneously as well as excellent reusability for at least three cycles. These results—achieved using oligomineral water with common interferons present in solution—highlight MOF-PES MMMs as efficient, scalable platforms for multimetal recovery, offering a sustainable pathway for LIB recycling.

## Graphical abstract

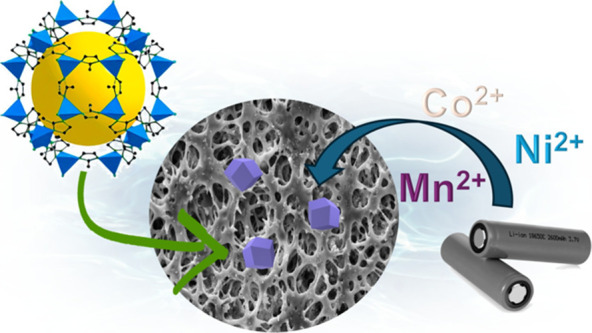

## Introduction

The global commitment to achieve carbon neutrality by 2050 has intensified the demand for sustainable energy production and the development of clean, renewable power sources to reduce reliance on fossil fuels and curb greenhouse gas (GHG) emissions.^1,2^ Among the various technologies supporting this transition, lithium-ion batteries (LIBs) have emerged as highly efficient energy storage systems, thanks to their high energy density, long cycle life, and proven scalability.^3^ Their widespread use in portable electronics, electric vehicles, and grid storage has driven an exponential increase in global demand, a trend expected to accelerate further in the coming decades.^4^ This growing market, however, presents a parallel challenge: the rapid accumulation of spent LIBs. By 2030, global waste from end-of-life LIBs is projected to exceed tens of millions of tons, including over four million tons of active cathode materials.^5^ Without proper treatment, this waste poses serious environmental and human health risks due to the toxicity of key components.^6,7^ Consequently, the sustainable recycling of LIBs has become a strategic priority—not only to mitigate environmental hazards but also to conserve critical resources.^8,9^ Spent LIBs are particularly attractive for recovery processes, as they contain lithium (Li)—and also other valuable metals such as cobalt (Co), nickel (Ni), or manganese (Mn)—often at higher concentrations than natural ores.^10^ Yet, meeting the rising global demand for these metals is complicated by limited mining capacity, geopolitical risks, and uneven distribution of reserves, underscoring the urgent need for efficient recovery technologies.

Current recycling strategies for metal ions from leachates include solvent extraction,^11–13^ chemical precipitation,^14^ adsorption,^15–18^ ion exchange,^19^ electrochemical processes,^20–22^ and biological methods.^23^ Among these, adsorption has shown notable promise due to its selectivity, stability, and energy efficiency. However, the selective and reversible capture of Co­(II), Ni­(II), and Mn­(II) from complex, multicomponent solutions remains a major challenge.^24^ Key research gaps include the identification of low-cost, environmentally friendly adsorbents with high capacity along with efficient regeneration and separation strategies.

Metal–organic frameworks (MOFs) are attractive candidates due to their large surface area, tunable porosity, and high water stability, which make them excellent platforms for removing organic and inorganic pollutants from water.^25–27^ Recent advances have focused on embedding MOFs into polymer matrices to create mixed matrix membranes (MMMs).^28,29^ These hybrid materials combine the robustness of polymers with the functionality of MOFs, yielding membranes with enhanced performance in separation processes and water treatment.^30–32^ Compared to MOF powders, MOF–MMMs are easier to process, mechanically more stable, and compatible with existing filtration platforms—addressing key barriers to the practical use of MOFs. Yet, optimizing their design to maximize the contribution of MOF fillers remains an open challenge.^33^

In a previous work, we demonstrated that MIL-53­(Al) incorporated into PES membranes achieved up to 95% removal efficiency for both Ni­(II) and Co­(II) ions in water.^34^ Building on this, the present study expands the scope to the simultaneous recovery of Mn­(II), Ni­(II), and Co­(II) cations from multicomponent solutions. Five MOFs—ZIF-8, MIL-53­(Al), and UiO-66, and their amino-functionalized analogues (NH_2_-MIL-53­(Al) and NH_2_–UiO-66)—were synthesized on a multigram scale, characterized, and incorporated into PES to fabricate MOF@PES MMMs. All MOF-based membranes outperformed pristine PES membranes, with ZIF-8@PES showing particularly high efficiency in the simultaneous adsorption of multiple heavy metal ions.

## Results and Discussion

Starting from our recent results^34^ that demonstrated the high removal efficiency of an MIL-53­(Al)-PES membrane for the adsorption of Ni­(II) and Co­(II) (up to 95%), we wanted to expand our investigation to other MOFs as adsorbent fillers. We selected some archetypal MOFs, based on their well-known water stability and structural robustness, adsorption affinity, pore structure, and dimension. All of the selected MOFs show high stability in water. ZIF-8 has relatively small pore windows but high surface area, suitable for the adsorption of metal ions. MIL-53­(Al) has larger pores, but its “breathing” behavior can help accommodate different ions. For all MOFs, interactions with Ni­(II), Co­(II), or Mn­(II) metal ions can be established with the oxygen atoms of the ligand for MIL-53 and UiO-66 or with the imidazolate ring of ZIF-8. Moreover, introducing −NH_2_ (amino) groups onto MOFs is a well-known strategy to improve metal ion adsorption.^35^ In particular, nitrogen-containing functional groups have been reported to enhance the adsorption capacity of MOFs for Co^2+^ and Ni^2+^.^36^ Amino groups can coordinate metal ions, acting as Lewis bases, thereby increasing the affinity, selectivity, and adsorption capacity. Moreover, the flexible and “soft” coordination of amino groups is likely to allow easy desorption during regeneration.

### Synthesis and Characterization of MOFs

A variety of well-known MOFs having different pore sizes and pore functional environments were selected and investigated for their adsorption properties toward the targeted metal ions. Five archetypical MOF—ZIF-8, MIL-53­(Al), and UiO-66, alongside their NH_2_-functionalized counterparts, NH_2_-MIL-53­(Al) and NH_2_–UiO-66)—were synthesized in a multigram scale, by following previously reported synthetic methods (see the Supporting Information).

The morphology of the polycrystalline samples was examined by using scanning electron microscopy (SEM). All the MOFs feature rather uniform particle size but different crystal sizes and shapes.

MIL-53­(Al) exhibited aggregates of small octahedral-shaped crystals of nanosized dimension (Figure 1a),^37^ while NH_2_-MIL-53­(Al) showed larger rectangular crystals (Figure 1b).^38^ Both UiO-66 and NH_2_–UiO-66 display regular octahedral-shaped crystals (Figure 1c,d), showing that the introduction of an amine functional group has a minor impact on the crystal morphology in this case.^39,40^ Finally, ZIF-8 showed crystals having a rhombic dodecahedron shape (Figure 1e).^41^

**Figure 1:**
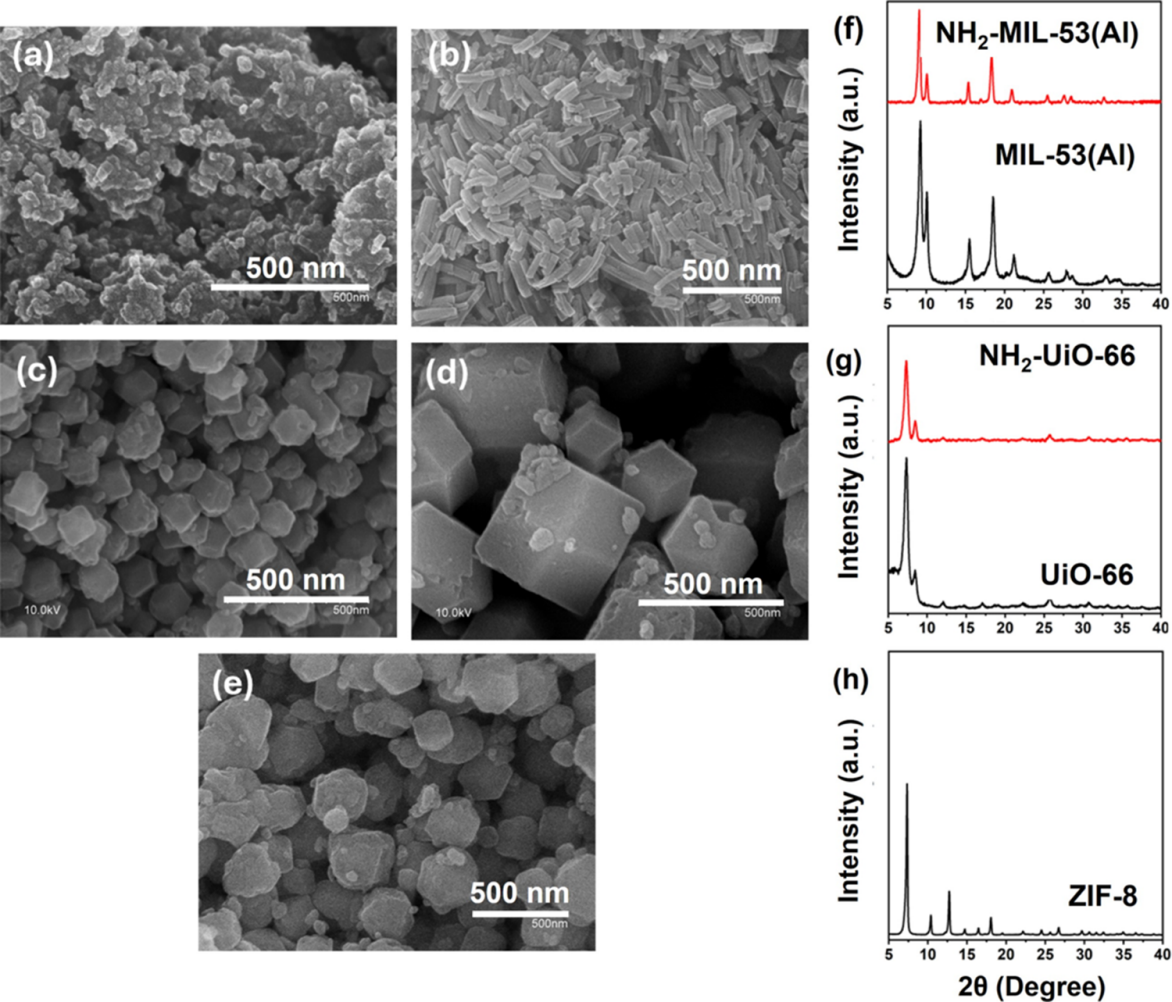
SEM images of different MOFs: (a) MIL-53­(Al), (b) NH_2_-MIL-53­(Al), (c) UiO-66, (d) NH_2_-UiO-66, and (e) ZIF-8 at 500 nm of magnification. XRD of *as-*synthesized MOFs (f) MIL-53­(Al) (black), NH_2_-MIL-53­(Al) (red), (g) UiO-66 (black), NH_2_–UiO-66 (red), and (h) ZIF-8.

Powder X-ray diffraction (XRD) was used to confirm the successful synthesis and the crystallinity of the MOFs. The XRD patterns of the functionalized MOFs were compared to those of their unfunctionalized counterparts. Results are presented in Figure 1f–h. The main peaks visible in the XRD patterns of MIL-53 (Al), NH_2_-MIL-53 (Al), UiO-66, and NH2-UiO-66 fit well with those reported in the literature.^42–45^ The samples are highly pure and crystalline, and no impurities are detectable. The crystal structure of the functionalized MOFs matches that of the pristine MOFs.

The specific surface areas and pore structures of the MOFs were characterized using Brunauer–Emmett–Teller (BET) analysis.^46^ The nitrogen adsorption–desorption isotherms and the distribution of pore sizes are displayed in Figure S1. The total surface area, pore size, and pore volume of each MOF are reported in Table 1. The obtained results showed substantial differences in the porosity of the synthesized MOFs. ZIF-8 displayed the highest specific surface area (1463.10 m^2^ g^–1^) and total pore volume (0.6389 cm^3^ g^–1^), and the smallest average pore width (0.77 nm). MIL-53­(Al) and NH_2_-MIL-53­(Al) showed similar average pore width (0.84 nm) and total pore volume (0.2078 cm^3^ g^–1^), but the amino-functionalized counterpart displayed a moderate decrease in surface area, with values of 491.63 m^2^ g^–1^ for NH_2_-MIL-53­(Al) and 895.64 for MIL-53­(Al), most likely due to the increase in the crystal size observed for the NH_2_-MIL-53­(Al) sample. A slighter reduction of the specific surface area was also observed for NH_2_–UiO-66 (737.44 m^2^ g^–1^) compared to UiO-66 (863.96 m^2^ g^–1^), while the total pore volume was rather preserved (0.3364 vs 0.3579 cm^3^ g^–1^, for NH_2_-UiO-66 and UiO-66, respectively).

**Table 1:** BET Surface Area and Pore Volume of Activated MOFs

MOFs	BET surface area (m^2^ g^–1^)	average pore width (nm)	total pore volume (cm^3^ g^–1^)
ZIF-8	1463.10	0.77	0.6389
MIL-53(Al)	895.64	0.84	0.2078
NH_2_-MIL-53(Al)	491.63	0.84	0.2078
UiO-66	863.96	0.91	0.3579
NH_2_-UiO-66	737.44	0.83	0.3364

### Preparation and Characterization of MOF-Based MMMs

MOF-based MMMs can offer very high adsorption capacities due to the presence of MOF adsorbent particles as filler—for example, the adsorption capacity of SU-101, a selective sorbent toward Ni^2+^ ions, reached 100.9 mg·g^–1^, while a near-zero adsorption capacity was found for Co^2+^ ions^47^—together with fast adsorption kinetics arising from their highly accessible porous structures. In comparison, ion-exchange resins can also provide high capacities for specific ions—an adsorption capacity as high as 139.60 mg/g was expected for Ni­(II)^48^—and exhibit rapid ion-exchange kinetics; however, their performance is often sensitive to ionic strength, competing ions, and water chemistry. Activated carbon generally shows moderate adsorption capacities (typically 150–300 mg g^–1^ for heavy metals, with an estimated maximum loading up to 400 mg g^–1^ for Ni­(II)^49^ but much lower experimental values^50^) with kinetics limited by internal pore diffusion. In terms of cost, MOFs remain the most expensive option due to synthesis-related expenses, while ion-exchange resins occupy an intermediate range, and activated carbon remains the lowest-cost alternative.

Owing to its favorable properties, PES was chosen as a suitable polymeric matrix for the preparation of MOF-based MMMs, while dimethyl sulfoxide (DMSO) was selected as a relatively less hazardous and less toxic solvent for this polymer.^34^ Neat PES and MOF-PES MMMs membranes have been prepared by nonsolvent-induced phase separation methods (NIPS) via phase inversion (see Scheme 1). To fabricate MOF-PES MMMs, homogeneous suspensions of MOF crystalline powders in DMSO were prepared. Each suspension was stirred slowly for at least 24 h until a uniform dispersion was achieved, and no particle aggregation was visible. Then, the dispersion was combined with a 25 wt % PES solution in DMSO and stirred for 24 h. The aimed MOF:PES ratio in the final membrane was 50:50 wt %. To produce a free-standing film with a nominal thickness of 250 μm, the solution was casted onto a glass substrate and immediately submerged in pure water used as a nonsolvent for the coagulation bath. The membranes were rinsed several times in water to remove any residual solvent trace and then preserved in ethanol. Net PES membranes were prepared following the same procedure but without the incorporation of MOFs.

**Scheme 1:**
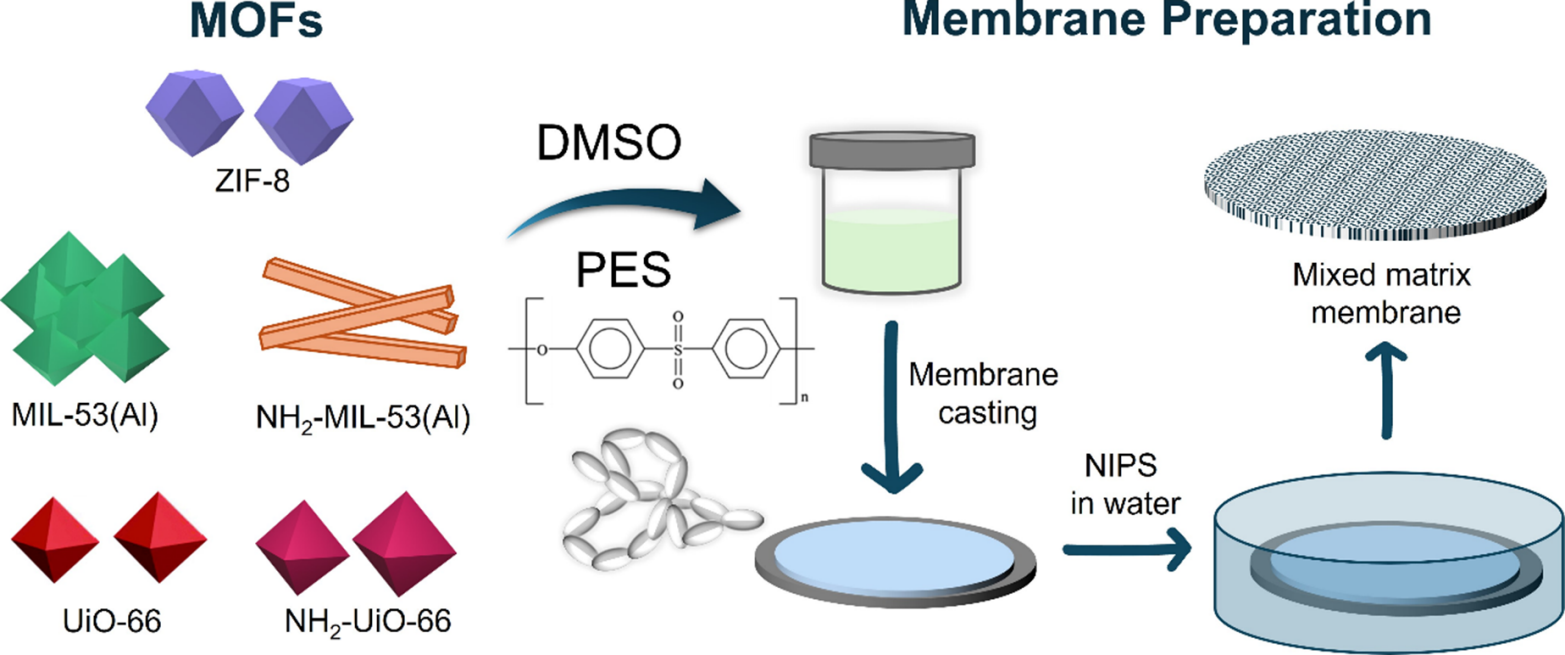
Schematic Representation of the Fabrication of MOF-PES MMMs

Although high MOF loadings are desirable to maximize the adsorption capacity of MMMs for the targeted application, they can also introduce drawbacks such as reduced mechanical strength and increased defect formation. These defects often arise from mismatches between the polymer matrix and filler particles or from particle aggregation.^51^ Remarkably, all of the MOF-PES MMMs in this study were highly flexible and pliable, showing no visible macroscopic defects and remaining deformable under stretching. The membranes were cut into circular discs (Figure 2), with an average thickness of 232 ± 2 μm and an active diameter of 46 ± 0.3 mm. The average mass was 48.5 ± 3 mg for pristine PES membranes and 72.25 ± 1 mg for MOF-loaded MMMs, corresponding to densities of 0.12 ± 0.02 and 0.17 ± 0.015 g cm^–3^, respectively.

**Figure 2:**
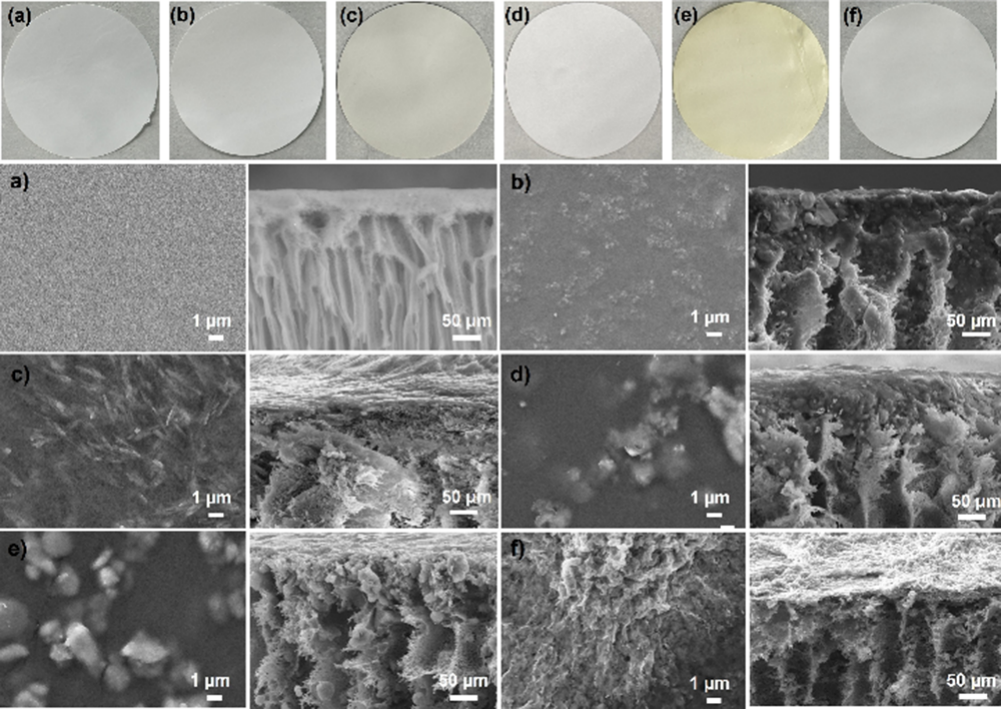
Top: Pictures of (a) PES, (b) MIL-53­(Al)-PES, (c) NH_2_-MIL-53­(Al)-PES, (d) UiO-66-PES, (e) NH_2_–UiO-66-PES, and (f) ZIF-8/-PES. Bottom: top (left) and cross-section (right) SEM images of (a) PES, (b) MIL-53­(Al)-PES, (c) NH_2_-MIL-53­(Al)-PES, (d) UiO-66-PES, (e) NH_2_–UiO-66-PES, and (f) ZIF-8-PES.

The influence of the MOF particles used as fillers on the morphology of the MMMs is illustrated in Figure 2 (bottom). All membranes exhibit a dense and thin top layer. However, the incorporation of MOF particles significantly modifies the internal membrane structure, transforming it from an essentially finger-like morphology to a hybrid structure that combines both finger-like and sponge-like features. This alteration is likely attributable to the hydrophilic nature of the MOFs, which promotes the formation of larger pores due to increased mass transfer between the solvent and nonsolvent phases during the phase inversion process.

The development of this hybrid structure enhances the stability of the MOF particles entrapped within the interlayered matrix, and it is likely to increase the accessibility to the active surface of the MOFs. In all membrane samples, some clusters and agglomerations of MOF particles are observed. This phenomenon can be attributed to the phase separation process during which the MOF and polymer components segregate into distinct regions within the membrane. The extent of this separation is influenced by their differing hydrophilicity and mutual affinity, leading to the formation of particle clusters within the membrane matrix.

FT-IR spectroscopy was performed on MOF powders and MMMs to prove the inclusion of MOFs within the membrane matrix (Figures S2–S4). PXRD was used to demonstrate the crystalline integrity of the MOFs after their incorporation into the polymer matrix. A prominent large peak at 2θ = 18° is observed for the pristine PES membrane, which is characteristic of the amorphous phase of the polymer (Figure 3a).^34^ The sharpness and intensity of the peaks in the MOF-PES MMMs confirm the high crystalline nature of the included MOFs (Figure 3b–f).^34,52,53^ A minor decrease in the peak intensity can be attributed to the dilution of MOFs in the amorphous polymeric matrix after the incorporation of the polycrystalline powder into the PES matrix. Nevertheless, the peaks almost maintain the same position for all MOF-PES MMMS, which further confirms that the crystallinity of MOFs is preserved upon inclusion in the polymer matrix.

**Figure 3:**
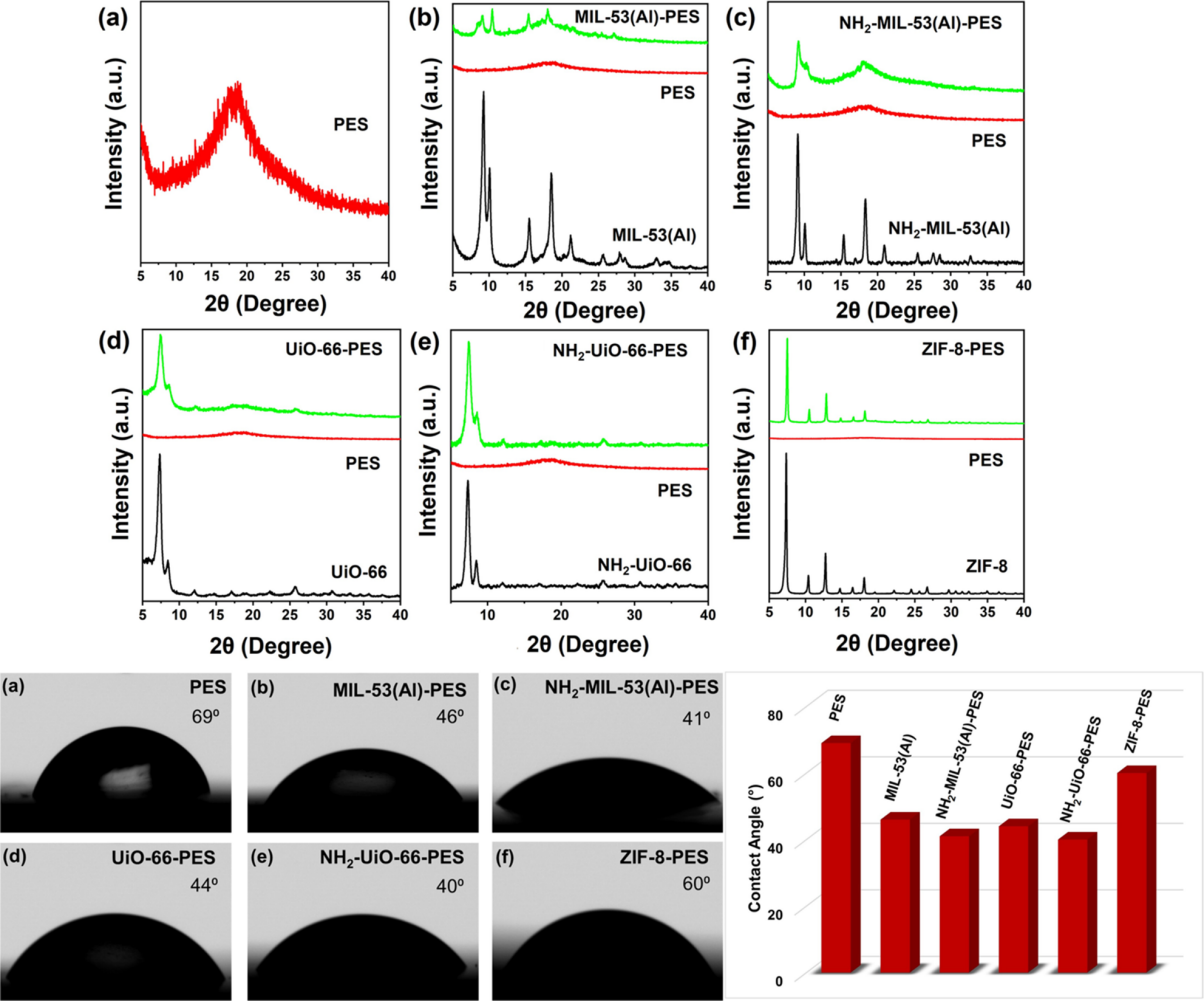
PXRD spectra (a–f top) and contact angle (a–f bottom) of (a) PES, (b) MIL-53­(Al)-PES, (c) NH_2_-MIL-53­(Al)-PES, (d) UiO-66-PES, (e) NH_2_-UiO-66-PES, and (f) ZIF-8-PES; and (bottom extreme right) comparison between the contact angle of all membranes.

Contact angle measurement was used as a suitable tool to evaluate the hydrophilic nature of the MMMs. Results are shown in Figure 3 (on the bottom) for different membrane surfaces. Pristine PES shows the highest contact angle, according to its reduced hydrophilic nature. Following the incorporation of the MOF into the PES matrix, the hydrophilicity of the PES membranes is significantly improved. ZIF-8-PES exhibits a lower hydrophilicity compared to other MOF-based membranes, while NH_2_-modified MOFs demonstrate the most significant reduction in contact angle, consistent with their higher hydrophilic character with respect to their parent MOFs.

### Mechanical Properties

The mechanical response of the membranes was evaluated in uniaxial tension at the STP (25 °C and 1 bar) using rectangular strips. For each composition, three specimens were tested. Young’s modulus (*E*) was estimated as the initial linear path of the stress–strain curve; yield strength (σ_y_) was determined by the offset method in accordance with ASTM D638;^54^ ultimate tensile strength, UTS (σ_ut_), and elongation at break (ε_f_) were read at the maximum stress at failure. The three repeats for each membrane produced highly repeatable curves, as reported in Figure 4. Results are listed in Figure 5, and the corresponding average values and standard deviations are summarized in Table 2.

**Figure 4:**
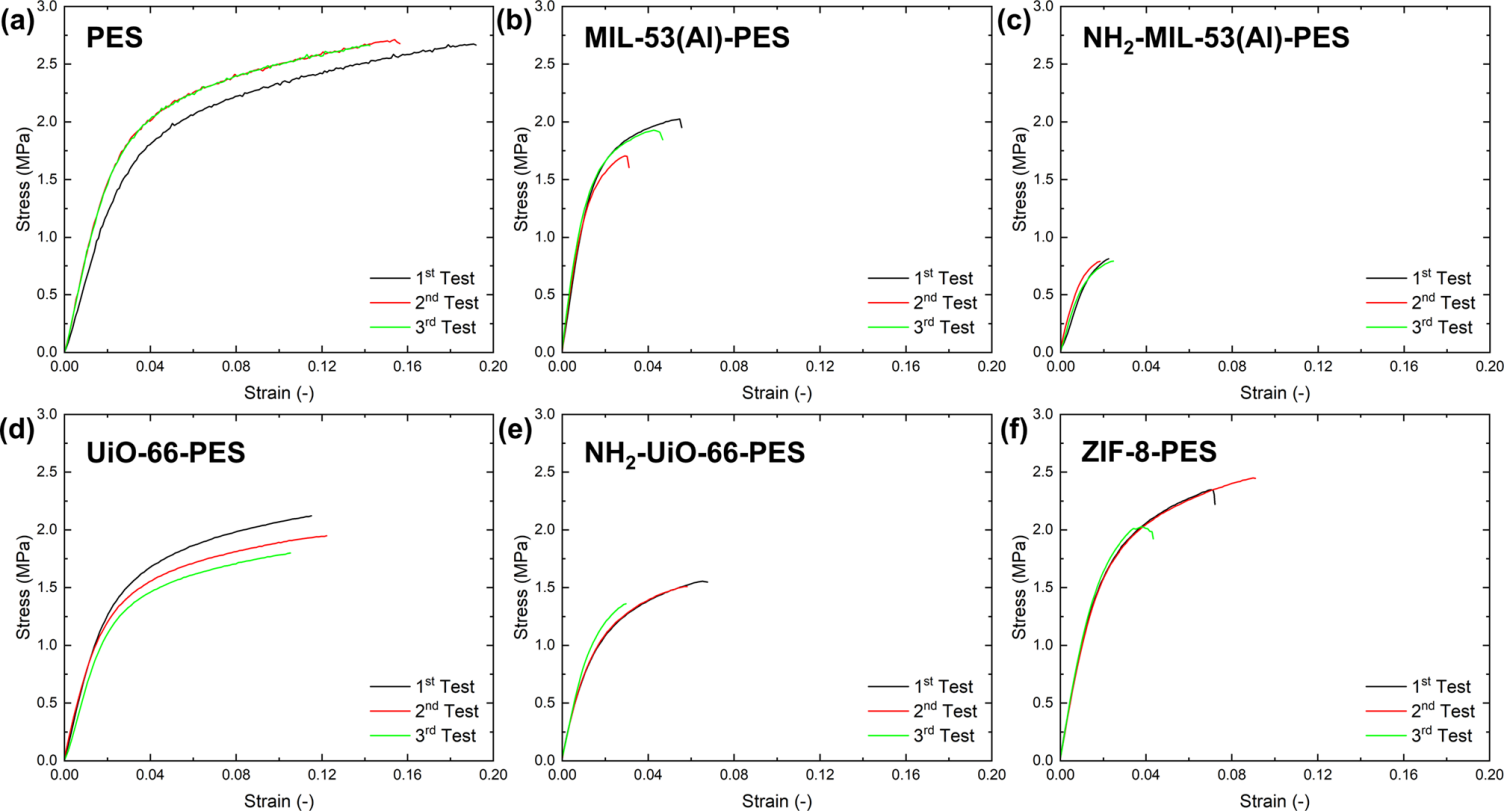
Measured engineering stress–strain (σ–ε) curves of investigated materials: (a) PES, (b) MIL-53­(Al)-@PES, (c) NH_2_-MIL-53­(Al)-PES, (d) UiO-66-PES, (e) NH_2_-UiO-66-PES, and (f) ZIF-8-PES.

**Figure 5:**
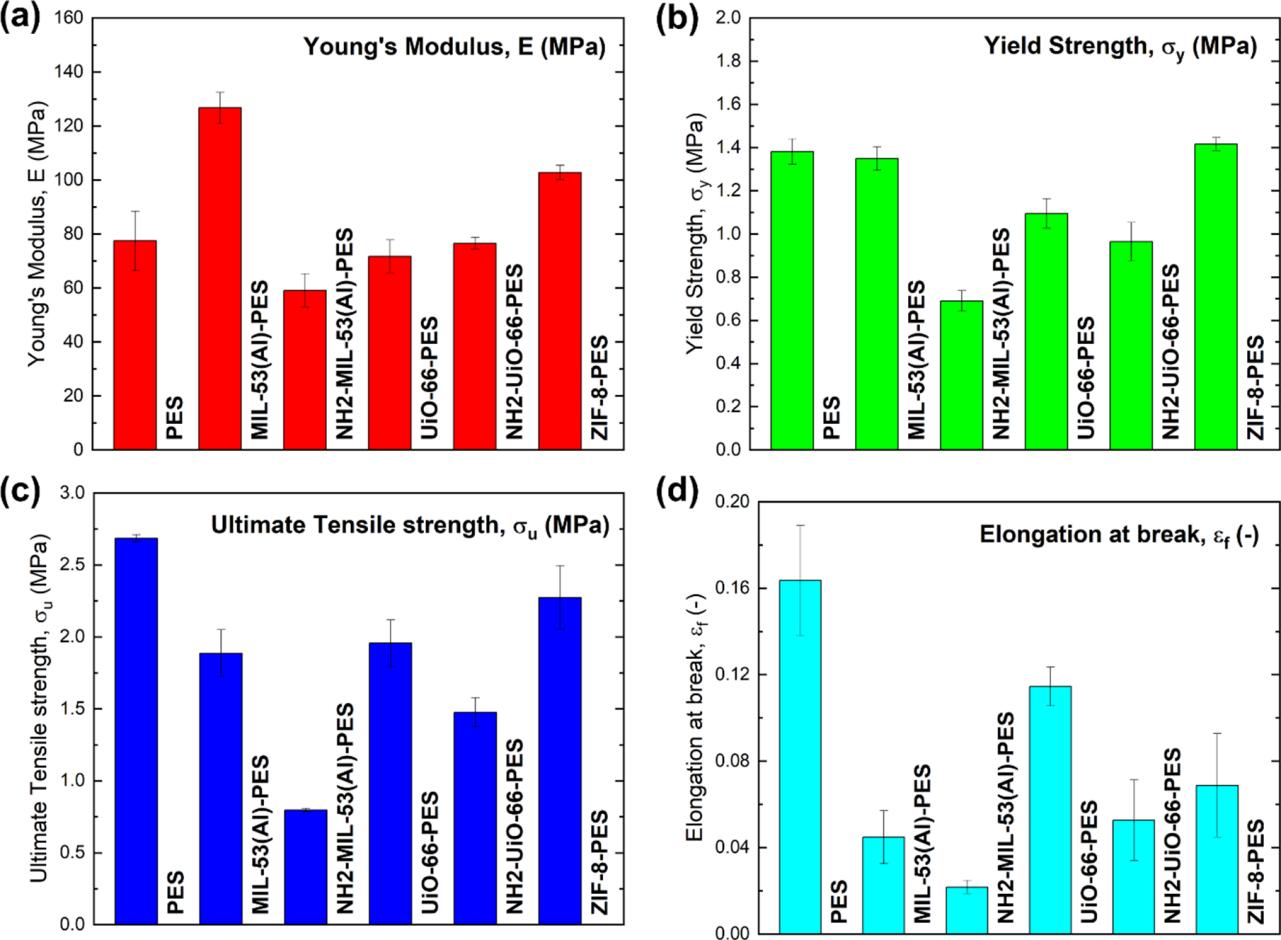
Comparison of significant macromechanical parameters between the investigated materials: (a) Young’s modulus (*E*), (b) yield strength (σ_y_), (c) ultimate tensile strength (σ_ut_), and (d) elongation to failure (ε_f_).

**Table 2:** Young’s Modulus, *E* (MPa), Yield Strength, σ_y_ (MPa), Ultimate Strength, σ_ut_ (MPa), and Elongation at Break, ε_f_ (−), of Different MOF-PES MMMs

membranes	Young's modulus *E* (MPa)	yield strength σ_y_ (MPa)	ultimate strength σ_ut_ (MPa)	elongation at break ε_f_ (−)
PES	77.5 ± 11.0	1.38 ± 0.06	2.68 ± 0.03	0.164 ± 0.026
MIL-53(Al)-PES	126.7 ± 5.8	1.35 ± 0.05	1.89 ± 0.16	0.045 ± 0.012
NH_2_-MIL-53(Al)-PES	59.1 ± 6.2	0.69 ± 0.05	0.80 ± 0.01	0.022 ± 0.003
UiO-66-PES	71.7 ± 6.1	1.09 ± 0.07	1.96 ± 0.16	0.115 ± 0.009
NH_2_-UiO-66-PES	76.5 ± 2.2	0.96 ± 0.09	1.47 ± 0.10	0.053 ± 0.019
ZIF-8-PES	102.7 ± 2.7	1.42 ± 0.03	2.27 ± 0.22	0.069 ± 0.024

Pristine PES exhibited a ductile response with a wide postyield region (*E* = 77.5 ± 11.0 MPa; σ_y_ = 1.38 ± 0.06 MPa; σ_ut_ = 2.68 ± 0.03 MPa; ε_f_ = 0.164 ± 0.026). Incorporation of MOFs affected the stiffness-ductility balance in a composition-dependent way, as shown in Figures 4 and 5. MIL-53­(Al)-PES provided the largest stiffening effect with respect to PES (*E* = 126.7 ± 5.8 MPa, +64%) with a slight reduction in the yield strength (σ_y_ = 1.35 ± 0.05 MPa, −2%) coupled with a marked reduction of UTS and elongation at break (σ_ut_ = 1.89 ± 0.16 MPa, −30%; ε_f_ = 0.045 ± 0.012, −73%). ZIF-8@PES also provided a stiffening effect (*E* = 102.7 ± 2.7 MPa, +33%) while largely preserving the yield point (σ_y_ = 1.42 ± 0.03 MPa, +3%) and retaining a relatively high UTS (σ_ut_ = 2.27 ± 0.22 MPa, −15%) with a moderate loss of ductility (ε_f_ = 0.069 ± 0.024, −58%). On the contrary, UiO-66-based membranes showed negligible stiffening effects with respect to PES, with a slight decrease in Young’s modulus (*E* = 71.7 ± 6.1 MPa, −7%), coupled with reduced strength and ductility (σ_ut_ = 1.96 ± 0.16 MPa, −27%; ε_f_ = 0.115 ± 0.009, – 30%). Similarly, NH_2_-UiO-66@PES showed no stiffening effects (*E* = 76.5 ± 2.2 MPa, −1%) combined with a marked reduction in the yield strength, UTS, and ductility (σ_y_ = 0.96 ± 0.09 MPa, – 30%; σ_ut_ = 1.47 ± 0.10 MPa, −45%; ε_f_ = 0.053 ± 0.019, −68%). The worst performance was obtained for NH_2_-MIL-53­(Al)@PES, which combined the lowest stiffness (*E* = 59.1 ± 6.2 MPa, −24%), with very low yield strength and UTS (σ_y_ = 0.69 ± 0.05 MPa, −50%; σ_ut_ = 0.80 ± 0.01 MPa, −70%) and a marked reduction in the elongation at break (ε_f_ = 0.022 ± 0.003, −87%). This resulted from premature failure caused by material embrittlement.

A comparative analysis among the studied fillers indicates that ZIF-8 and MIL-53­(Al) particles provided the highest stiffening effects but at the cost of a reduced ductility. This can be linked to a reduced chain mobility, stress-transfer to stiff inclusions, and defect/void formation, thereby limiting the load-bearing capability at high strain. In addition, ZIF-8 provided the best trade-off between stiffness and strength retention, whereas amino-functionalized MIL-53­(Al) severely degraded all mechanical properties, likely reflecting lower interfacial adhesion and/or larger defect density during phase inversion. These trends are consistent across the three replicates per formulation and are evident in both the averaged metrics (Table 2) and the stress–strain curves (Figure 4).

The maximum practical MOF loading for these systems was determined to be 50 wt % based on our optimization experiments.^34^ This load was selected because it provides the best compromise between achieving high adsorption capacity and maintaining sufficient mechanical integrity for the intended operating conditions. Importantly, the targeted application—microfiltration for metal-ion recovery—operates at low pressures (<1 bar), for which the mechanical strength of the 50 wt % MMMs is adequate.

For applications requiring higher mechanical robustness or operation at elevated pressures, a viable next step would be to fabricate composite membranes in which the selective MMM layer is deposited onto a highly porous support. This configuration is commonly employed to enhance the overall mechanical stability while preserving the functional advantages of the MOF-loaded active layer.

### MOF Performance for Ni­(II), Co­(II), and Mn­(II) Single Metal-Ion Adsorption

Before evaluating the performance of MOF-PES MMMs, we examined the efficiency of MOFs as polycrystalline powders in removing Ni­(II), Co­(II), and Mn­(II) cations from water as the main component of LIBs. The chosen metal ratio (1:1:3 for Ni:Mn:Co) corresponds to the proportion of these metals usually found in an actual waste sample (see Table S2).

Lithium was not included in the present study because its adsorption using conventional MOFs is intrinsically difficult. Li^+^ is a very small, strongly hydrated cation, and most MOFs do not provide interaction sites capable of competing with its hydration shell.^55^ The effective hydrated diameter of Li^+^ (∼7–8 Å) also prevents its diffusion through the small pore apertures of many widely studied MOFs such as ZIF-8 (∼3–4 Å). Furthermore, Li^+^ behaves as a hard Lewis acid and therefore interacts weakly with the neutral or N-donor binding sites present in the MOFs used here, including amino-functionalized structures. Although targeted functionalization with hard O-donor groups (e.g., −O^–^, −COO^–^, and −PO_4_^3–^) could improve lithium binding, such groups would strongly compete with other multivalent ions typically present in LIB leachates. Future work will explore MOFs with tailored pore sizes and specific hard-donor functionalities toward Li^+^ recovery, potentially enabling these MMMs to complement complete LIB recycling schemes.

Adsorption experiments were carried out by soaking 20 mg of each MOF for 72 h in 10 mL of an aqueous solution prepared with an oligomineral water containing common interfering ions (Table S1) and the target metal ions, at initial concentrations of 200 mg/g (400 mg/L) for Ni­(II) and Mn­(II) and 600 mg/g (1200 mg/L) for Co­(II), respectively.

We collected small aliquots of the multi-ion solution after 3 days and analyzed the residual metal content using ICP-MS in order to calculate the equilibrium maximum loading (*Q*_e_) and the removal efficiency *R*(%) of each MOF.

The calculated removal efficiency *R*(%) and maximum loading are shown in Figure 6 (Table S3). The equilibrium maximum loading was calculated by using eq 1.

$$Q_{e}=\frac{(C_{0}-C_{e})\times V}{m}$$ 1

where *C*_0_ is the initial concentration of metal ions in the solution (mg/L), *C*_e_ is the equilibrium concentration of metal ions in the solution (mg/L), *V* is the volume of the solution (L), and *m* is the mass of the MOF powder (g).

**Figure 6:**
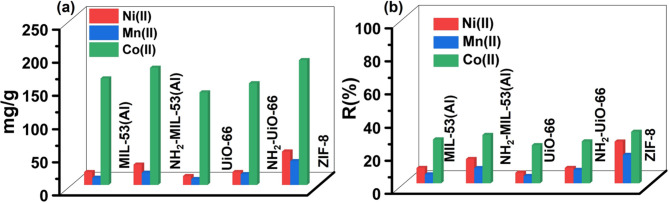
(a) Equilibrium maximum loading and (b) removal efficiency determined by soaking 20 mg of the polycrystalline samples of the selected MOFs (from the left: MIL-53­(Al), NH_2_-MIL-53­(Al), UiO-66, NH_2_-UiO-66, and ZIF-8) in 10 mL of an aqueous solution of mineral water containing common interfering ions and the target metal ions (initial concentrations of 200 mg/g for Ni­(II) and Mn­(II) and 600 mg/g for Co­(II) (graphics are organized from data reported in Table S3). Each experiment was performed in triplicate, and results are reported as average values ± 3 SD.

The removal efficiency was calculated according to eq 2:

$$R(\%)=\frac{\left(C_{0}-C_{e}\right)}{C_{0}}$$ 2

where *C*_0_ is the initial concentration of the metal ions in the solution, and *C*_e_ is the equilibrium concentration after adsorption.

In general, all MOFs demonstrate promising performance for metal-ion removal. As clearly shown in Figure 6, ZIF-8 has the highest removal efficiency and adsorption capacity compared to the other MOFs for all metal cations. Specifically, the highest loading values have been obtained for Co­(II) ions (*R*(%) = 31.2 and *Q*_e_ = 188.0 mg/g), followed by Ni­(II) ions (*R*(%) = 25.3% and *Q*_e_ = 50.5 mg/g) and Mn­(II) (*R*(%) = 17.2 and *Q*_e_ = 35.9 mg/g). MIL-53­(Al) demonstrates moderate adsorption and removal efficiency for the targeted ions, with higher values compared to UiO-66 for all of the metal species. Overall, NH_2_-functionalized MOFs (NH_2_-MIL-53­(Al) and NH_2_-UiO-66) showed improved performance in terms of removal efficiency and equilibrium maximum loading for all the metal ions as compared to their parent MOFs (MIL-53­(Al) and UiO-66), despite their slightly reduced BET surface and total pore volume. These results indicate an increased affinity for these metal ions, likely due to the optimized and more suitable pore environment created by the NH_2_ functional groups.^36^

Overall, the observed removal efficiency and selectivity result from the interplay between porosity (surface area, pore volume, pore architecture) and chemical functionality (imidazolate rings, −NH_2_ groups, and linker heteroatoms), which together govern both the accessibility of adsorption sites and the strength of metal–ligand interactions, as well as by the nature of the adsorbed metal ions.

The higher removal efficiency of ZIF-8-PES arises from the combination of its structural and chemical features. Among the synthesized MOFs, ZIF-8 exhibits the largest BET surface area (1463.1 m^2^ g^–1^) and pore volume (0.6389 cm^3^ g^–1^) compared to MIL-53­(Al) and UiO-66, together with a small, well-defined pore aperture (Figure S1) and high crystallinity (Figure 3). These characteristics provide a greater number of accessible adsorption sites for Co^2+^, Ni^2+^, and Mn^2+^. In addition, the chemical environment of ZIF-8—particularly the imidazolate ring—promotes strong interactions with the target metal ions, as also discussed in the literature.^36^ Concerning MIL-53­(Al), although having a lower surface area, it benefits from its flexible ‘breathing’ pore environment, which facilitates ion accommodation.

For the amino-functionalized MOFs (NH_2_-MIL-53­(Al) and NH_2_–UiO-66), the – NH_2_ groups confirmed by FT-IR (Figure S2) slightly reduced surface areas but introduced additional coordination sites for metal ions, thereby enhancing affinity and selectivity, particularly for Co^2+^ and Ni^2+^. This is consistent with the reported behavior of N-functionalized MOFs, where metal adsorption occurs through coordination to amine groups as well as to heteroatoms of the organic linker and agrees with the results reported in a recent research work, which demonstrated that the introduction of N-containing groups in an amino-modified MOF (MIL-101-NH_2_) enhanced the adsorption capacity for both Co^2+^ and Ni^2+^ ions.^36^

The observed selectivity trend (Ni^2+^ ≈ Co^2+^ > Mn^2+^) and the differences in metal-ion affinity among Ni^2+^, Co^2+^, and Mn^2+^ can be rationalized by the Irving–Williams series and ligand-field stabilization energies by considering the ionic size, hydration behavior, and compatibility with the MOF pore environment. Ni^2+^ (0.69 Å) and Co^2+^ (0.75 Å) possess smaller ionic radii and lower hydration diameters than Mn^2+^ (0.80 Å), enabling easier partial dehydration and diffusion into the MOF pores. Their higher charge density and stronger ligand-field stabilization also promote a more stable coordination with the N-donor sites of ZIF-8 and the O-donor sites of MIL-53­(Al) and UiO-66.

Furthermore, Ni^2+^ forms intrinsically more stable complexes than high-spin Co^2+^ and Mn^2+^ ones and has the highest electronegativity (1.3678 eV),^56^ favoring stronger interactions with electron-donating sites such as −NH_2_ groups or linker N/O atoms. In contrast, the larger hydrated Mn^2+^ ion experiences weaker binding and less efficient pore accommodation.

These factors collectively explain the consistently higher uptake of Ni^2+^ and Co^2+^ observed across the MOFs (with the Co^2+^ performance also being influenced by its higher initial concentration). Taken together, these steric and thermodynamic factors are also expected to drive the preferential adsorption of Ni^2+^ and Co^2+^ over Mn^2+^ across all MOF-PES membranes.

### MOF-PES MMMs for the Simultaneous Capture of Ni­(II), Co­(II), and Mn­(II)

We evaluated the performance of the pristine PES membranes and the MOF-PES MMMs by performing batch experiments for the recovery of the three target metal ions from an oligomineral aqueous solution containing 1 ppm of Ni­(II) and Mn­(II) and 3 ppm of Co­(II), as well as common interfering metal ions (Table S1).

The simultaneous and effective capture of different metal ions in the presence of common interfering species represents a groundbreaking step toward water remediation and precious metal recovery. The removal efficiency *R*(%) and equilibrium maximum loading (*Q*_e_) were calculated over time using ICP-MS (Inductive Couple Plasma Mass Spectroscopy) to measure the residual metal concentration in the solution.

As reported in Figure 7 (Tables S4–S6), the pristine PES membrane exhibits a reduced adsorption capacity compared to the MOF-PES MMMs, underlying the important role of the MOF porous material used as a filler. The presence of different MOFs with distinctive characteristics modulates the affinity of the membrane for the targeted metal ions. ZIF-8-PES membranes consistently showed the highest adsorption capacity and removal efficiency for all three ions, reaching *R*(%) values higher than 90% (Table S7), and residual metal concentrations in the range of 0.07–0.3 ppm. In particular, the residual concentrations of the two more concerning metal ions, i.e., Ni­(II) and Mn­(II), reach values of 80 and 70 μg/L, respectively, which are close to or lower than the acceptable limits established by WHO for drinking water (Ni­(II) 70 μg/L and Mn­(II) 80 μg/L),^57,58^ (for cobalt ions, there are no specific guideline values in WHO standards). A good degree of selectivity was observed for MIL-53­(Al)-PES, which preferentially adsorbed Co­(II) and Ni­(II) ions (*R*(%) 97% for Co­(II) and 91% for Ni­(II)) over Mn­(II) (*R*(%) slightly above 30%) (Table S7).

**Figure 7:**
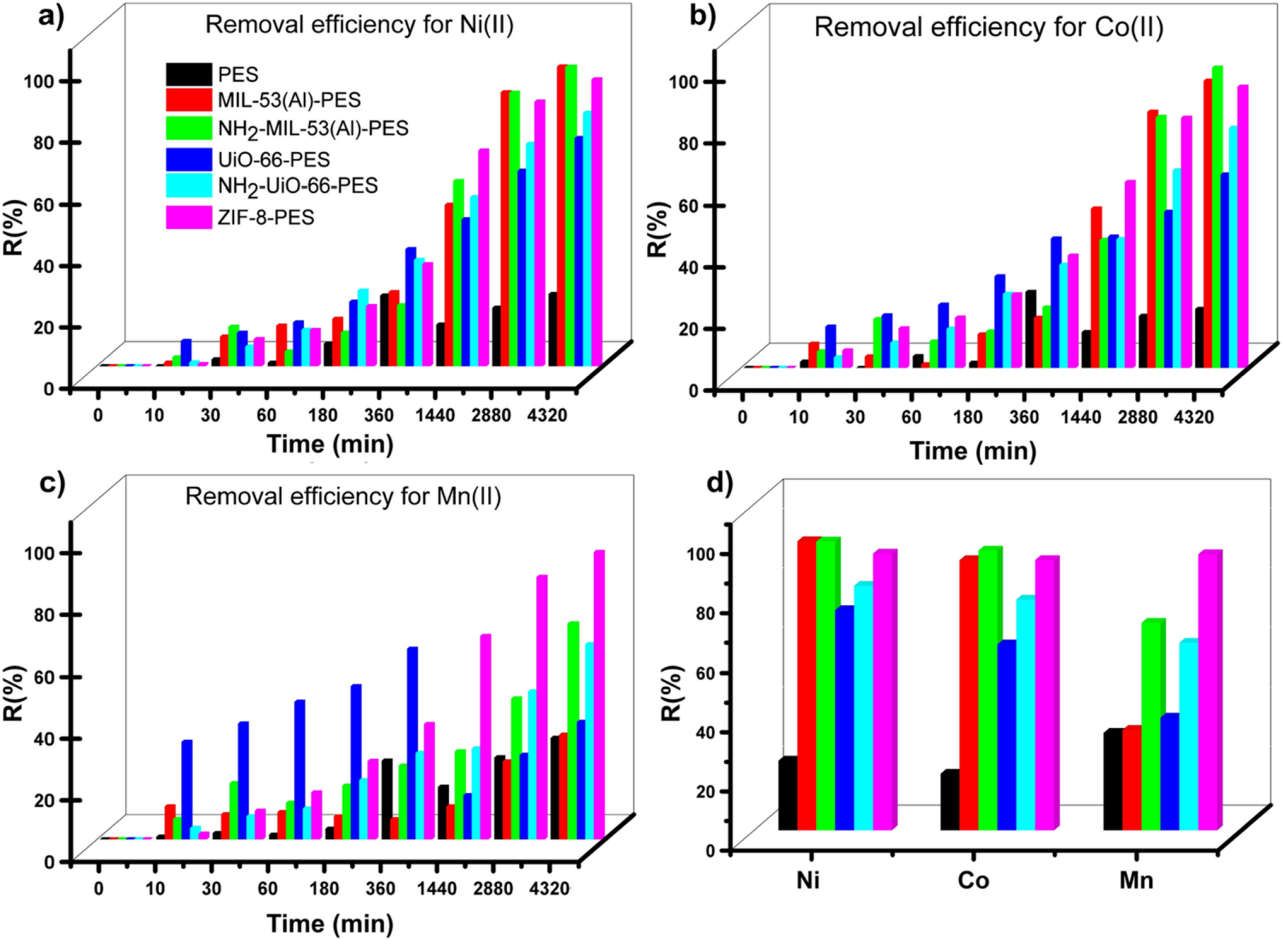
Removal efficiency for Ni­(II) (a), Co­(II) (b), and Mn­(II) (c) target ions determined by soaking MOF-PES MMMs in 100 mL of a mineral aqueous solution containing 1 ppm of Ni­(NO_3_)_2_·6H_2_O, 2 ppm of Co­(NO_3_)_2_·6H_2_O, and 1 ppm of Mn­(NO_3_)_2_·4H_2_O. (d) Summary of the maximum removal efficiencies by the different materials [PES (black), MIL-53­(Al)-PES (red), NH_2_-MIL-53­(Al)-PES (green), UiO-66-PES (blue), NH_2_-UiO-66-PES (cyan), and ZIF-8-PES (purple)]. Each experiment was performed in triplicate, and results are reported as average values ± 3 SD (the experimental data can be found in Tables S4–S6).

As already observed for the MOFs, the introduction of the amino groups in both MIL-53­(Al) and UiO-66 enhances the adsorption capacity and removal efficiency of MMMs for all three ions, with the NH_2_-MIL-53­(Al)-PES membranes showing better performances with respect to NH_2_–UiO-66-PES (*R*(%) > 97% for Ni­(II) and Co­(II) and >70% for Mn­(II)). The enhanced ability of these two membranes, i.e., ZIF-8-PES and NH_2_-MIL-53­(Al)-PES, to simultaneously remove Ni­(II)/Co­(II)/Mn­(II) or Ni­(II)/Co­(II) ions can be attributed to the characteristics of the employed MOFs, including their high surface areas and optimized pore structures and functionalization, underlying the importance of surface chemistry in metal ion capture.

### Stability and Regeneration of MOF-PES MMMs

The stability and reusability of the three best-performing MOF-PES MMMs (MIL-53­(Al)-PES, NH_2_-MIL-53­(Al)-PES, and ZIF-8-PES) were determined since both parameters are essential for both their application in real environments and their upscale. The MOF-PES MMMs were soaked in a 4:1 H_2_O/CH_3_CH_2_OH mixture for 1 day to remove the already captured metal ions and then further utilized for additional adsorption tests.

To quantify the recovery process, we conducted three consecutive adsorption-desorption cycles and analyzed the solutions after each step using ICP-MS. This allowed us to determine the desorption efficiency (metal recovery) and the removal efficiency for Mn^2+^, Co^2+^, and Ni^2+^ after each cycle. The metal recovery percentage was calculated using eq 3:

$$\mathrm{MR}(\%)=\frac{\left(C_{d}\right)}{\left(C_{0}\right)}$$ 3

where *C*_d_ is the concentration of the desorbed metal, and *C*_0_ is the initial metal concentration. After three cycles of reuse, all of the membranes, ZIF-8-PES, MIL-53­(Al)-PES, and NH_2_-MIL-53­(Al)-PES, showed very good reusability, demonstrating the stability and robustness of the membranes (Tables S8 and S9 and Figure 8). In particular, ZIF-8-PES membranes constantly showed the highest adsorption capacity and removal efficiency for all three ions, with *R*(%) values higher than 90 after three cycles of reuse (Table S8).

**Figure 8:**
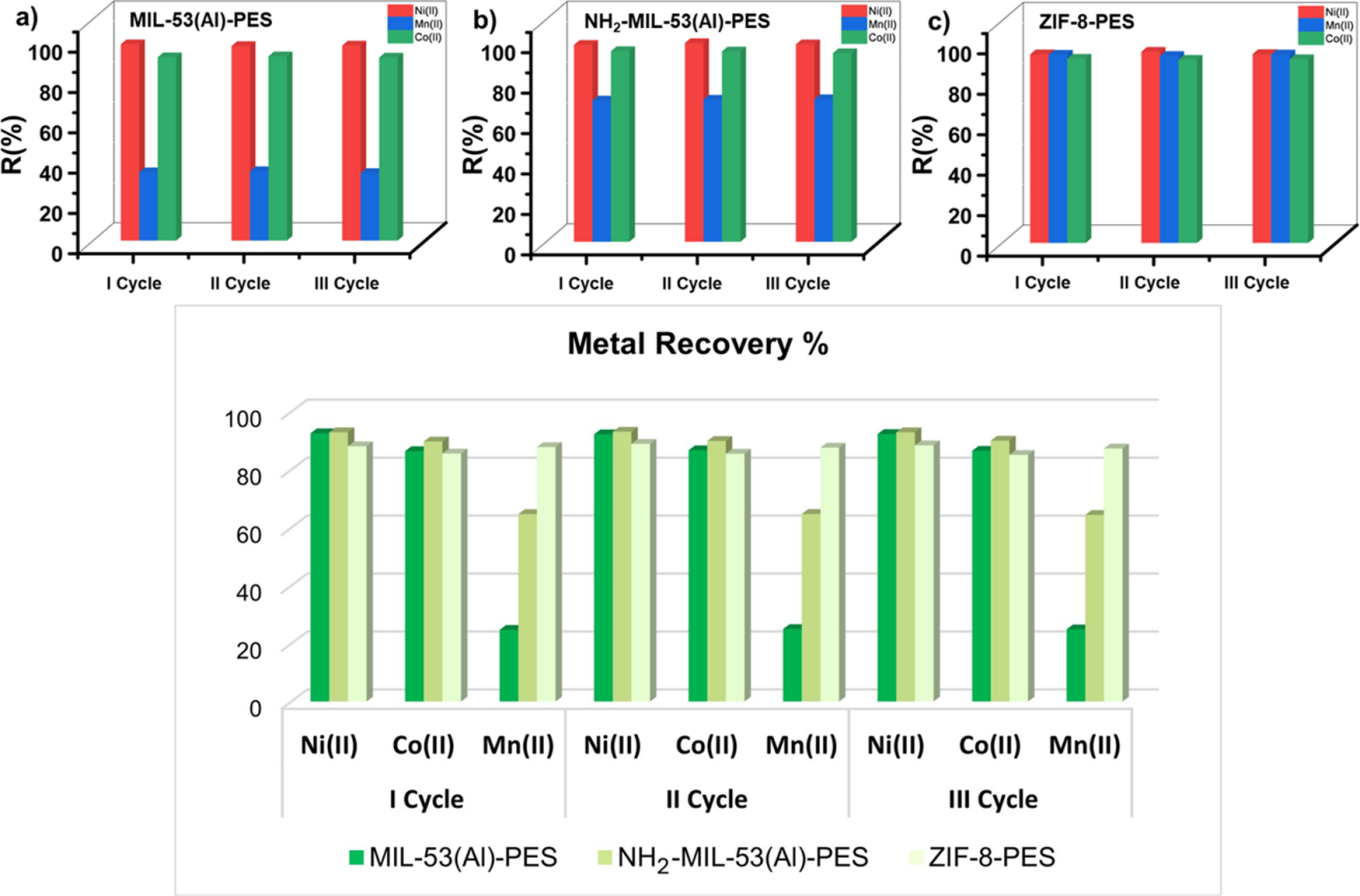
Reuse of the MOF-PES MMMs [MIL-53­(Al)-PES, NH_2_-MIL-53­(Al)-PES, and ZIF-8-PES] soaked in a 4:1 H_2_O/CH_3_CH_2_OH mixture for 1 day to remove the already captured metal ions and then further utilized for the adsorption of Ni­(II), Mn­(II), and Co­(II) target ions. Top: Removal efficiency for (a) MIL-53­(Al)-PES, (b) NH_2_-MIL-53­(Al)-PES, and (c) ZIF-8-PES. Bottom: Metal recovery after desorption for MIL-53­(Al)-PES, NH_2_-MIL-53­(Al)-PES, and ZIF-8-PES.

PXRD and SEM analyses of the membranes after three regeneration cycles have also been collected to assess the membrane stability (Figures S5 and S6). PXRD patterns show that the characteristic diffraction peaks of the MOFs are retained, confirming that the crystalline structure of the MOF remains intact after repeated use. Likewise, SEM images of both the membrane surface and cross-section indicate that the MOF particles remain well dispersed within the PES matrix, with no observable aggregation or leaching. These structural observations are fully consistent with the stable adsorption performance measured across the regeneration cycles.

Although MOFs can be relatively costly,^58^ the amount incorporated into each membrane is small, and their reusability over several adsorption-desorption cycles significantly reduces the effective operational cost. When compared with the market value of the recovered Ni, Co, and Mn—particularly cobalt—the recovery benefit can partially offset membrane fabrication costs. Thus, the combination of reusability and metal recovery value suggests that MOF@PES MMMs offer a promising and potentially sustainable option for LIB recycling processes.^59^

Moreover, recent developments in sustainable metal recovery highlight the need for low-energy circular approaches to treat complex waste streams. For example, multimetal extraction from photovoltaic wastes^60^ and emerging electrolyte-reuse strategies in electronic-waste recycling demonstrate viable pathways for scalable, resource-efficient metal recovery. Within this growing landscape, our MOF-PES MMMs provide a complementary, mildly operating option for simultaneous capture of multiple critical metals.

The scalable fabrication of MOF-PES MMMs via solution casting/NIPS and polymer-supported MOFs enables their easy integration into modular water treatment and LIB recycling frameworks. Membrane-based processes offer low energy consumption, minimal chemical input, and compact, flexible designs that are suitable for continuous operation. Parallel cartridges or stacked modules can be employed for selective preconcentration of metal ions prior to downstream solvent extraction or electrochemical recovery. The mild, roll-to-roll compatible fabrication and chemical stability of these membranes support repeated reuse, while renewable energy sources, such as photovoltaic-driven filtration units, can be coupled to further enhance sustainability. This approach positions MOF@PES MMMs as a practical, modular, and energy-efficient solution for industrial-scale metal recovery and wastewater remediation.^61^ In addition, scalable synthesis routes have been established for each of these MOFs, which strengthens their prospects for use in real-world applications.

A brief life-cycle perspective also highlights the environmental advantages of adsorption–microfiltration processes, such as those used in this study. Unlike vacuum distillation or hydrometallurgical refining—both known for high energy demand, extensive solvent consumption, and concentrated waste generation—MOF-based separations operate under mild conditions, typically at ambient temperature and without harsh reagents. Recent studies have shown that MOF-based systems can offer lower embodied energy and reduced environmental burdens compared with conventional separation technologies, particularly when immobilized in continuous-flow configurations that improve reusability and reduce chemical inputs.^62^ Although a full life-cycle assessment is beyond the scope of this work, our MOF-PES membranes align with sustainable separation frameworks by minimizing energy use, solvent requirements, and secondary waste while enabling recyclable operation.

## Conclusions

In this work, we investigated the adsorption of Co^2+^, Ni^2+^, and Mn^2+^ ions onto MOF-PES MMMs incorporating five different MOFs: ZIF-8, MIL-53­(Al), and UiO-66, along with their amino-functionalized derivatives, NH_2_-MIL-53­(Al), and NH_2_–UiO-66. Among them, ZIF-8-PES membranes exhibited the highest adsorption capacity and removal efficiency (>90%) for the simultaneous capture of all three metal ions. MIL-53­(Al)-PES showed moderate selectivity, preferentially adsorbing Co^2+^ and Ni^2+^ over Mn^2+^. Notably, amino functionalization enhanced the affinity of both MIL-53­(Al) and UiO-66 for all target ions, significantly improving their adsorption performance.

A clearer interpretation of the selectivity of the different MOFs toward Ni^2+^, Co^2+^, and Mn^2+^ can be established by correlating their porosity and pore chemistry. ZIF-8, with the highest surface area and pore volume, provides more accessible adsorption sites, whereas MIL-53­(Al) benefits from its flexible pore structure and UiO-66 from its rigid, highly connected framework. Amino-functionalized MOFs additionally introduce −NH_2_ groups that serve as strong Lewis base sites, enhancing coordination with Ni^2+^ and Co^2+^. The observed affinity order (Ni^2+^ > Co^2+^ > Mn^2+^) follows the Irving–Williams series and reflects intrinsic metal–ligand stability: Ni^2+^, being smaller and more electronegative, forms more stable interactions with oxygen- and nitrogen-donor sites. Overall, selective adsorption arises from the combined influence of the surface area, pore environment, and functional groups.

To assess practical applicability, adsorption experiments were conducted with solutions containing Ni^2+^, Co^2+^, and Mn^2+^ in concentrations and ratios representative of LIB leachates and mineral water containing common interfering ions. The MOF-PES membranes maintained high efficiency under these realistic conditions. Furthermore, stability tests demonstrated that the membranes could be reused at least three times without significant loss of performance. These findings highlight the robustness and potential of MOF@PES MMMs for the removal and recovery of critical metal ions from complex aqueous environments. The superior performance of ZIF-8-PES for simultaneous Ni^2+^/Co^2+^/Mn^2+^ removal and of NH_2_-MIL-53­(Al) for selective Ni^2+^/Co^2+^ capture is attributed to their large active surface area, suitable pore architecture, and favorable surface chemistry. Overall, the results underscore the importance of judicious MOF selection and pore functionalization in the design of efficient membrane materials for ion adsorption and separation.

In summary, the novelty of this work lies in the integrated and systematic exploration of functionalized MOF-PES MMMs for the simultaneous recovery of Ni^2+^, Co^2+^, and Mn^2+^ from realistic, multicomponent solutions, along with mechanistic insights and reusability assessment. This advances membrane-based resource recovery beyond single-ion studies and contributes new understanding relevant to LIB recycling processes.^63,64^

## Supplementary Material



## Notes

Experimental details, including characterization images and metal recovery data; Tables S1–S9; and Figures S1–S6 (PDF)
