# Supplementary material for: Membranes Based on Metal–Organic Framework Nanostructures for Recovering Nickel, Cobalt, and Manganese Ions from Spent Lithium-Ion Batteries
Source: ACS Appl Nano Mater. 2025 Dec 18;9(1):424–36. doi: 10.1021/acsanm.5c04698 (PMC12797191; doi:10.1021/acsanm.5c04698)
Supplement: Supplementary file 1 [file an5c04698_si_001.pdf]

Supporting Information

Membranes Based on Metal-Organic Framework-Nanostructures for Recovering Nickel, Cobalt, and Manganese Ions from Spent Lithium-Ion Batteries.

Waseem Iqbal, ‡[a] Amira Nour, ‡[a] Rosaria Bruno,[a] Pietro Magarò,[b] Carmine Maletta,[b] Rosangela Elliani,[a] Antonio Tagarelli,[a] Teresa F. Mastropietro*,[a] Jesús Ferrando–Soria,[c] Emilio Pardo*,[c] and Donatella Armentano*,[a]

[a] Dipartimento di Chimica e Tecnologie Chimiche, Università della Calabria. 87030, Rende, Cosenza, Italy.

[b] Dipartimento di Ingegneria Meccanica, Energetica e Gestionale, Università della Calabria. 87030, Rende, Cosenza, Italy.

[c] Instituto de Ciencia Molecular (ICMOL). Universitat de València Paterna 46980, València (Spain)

email: donatella.armentano@unical.it, emilio.pardo@uv.es, teresafina.mastropietro@unical.it

Experimental Section.

Preparation of MIL-53(Al) and NH_2-MIL-53(Al).

Typical solvothermal synthesis was used for the preparation of MIL-53(Al) and NH_2-MIL-53(Al).[1] Solution A was prepared by weighing aluminium(III) nitrate nonahydrate ($Al(NO3)_3 \cdot 9H_2O$) (1.12 g, 2.98 mmol) and placing it in 15 mL of N,N-dimethylformamide (DMF). Solution B was done by adding 1,4-benzenedicarboxylic (BDC) acid (1.11 g, 6.68 mmol) in 15 mL DMF. Each solution was left under continuous stirring for 1 h. Afterward, metal solution was added dropwise to the ligand, and the resulting solution was left under stirring at 300 rpm for 20 min at room temperature (RT). The mixture was then transferred to a 56 mL Teflon-lined hydrothermal autoclave. Finally, the oven was used to heat the sample to 120 °C for 72 h. Subsequently, the Teflon-lined autoclave gradually cooled down to room temperature. Then, the solid phase was separated using centrifugation (HERMLE) at 6000 rpm and was washed three times with DMF and MeOH to remove the unreacted terephthalic acids trapped in the pores of the obtained product. The MOF was dried under vacuum overnight. The product was a solid white powder, which was activated at 70 °C for 12 h and was subjected to different analyses for identifying and ensuring the correct formation of MIL-53(Al). The same procedure was utilized for the preparation of NH_2-MIL-53(Al), where terephthalic acid was replaced by 2-amino terephthalic acid (1.74 g, 6.68mmol).

Preparation of UiO-66 and NH_2-UiO-66.

The preparation of UiO-66 and NH_2-UiO-66 was carried out following previous research.[2-4] 0.53 g of zirconium (IV) chloride ($ZrCl_4$) and 0.38 g of terephthalic acid were dissolved in 30 mL of DMF and stirred vigorously until a clear and homogeneous solution was obtained. The homogeneous solution was placed in a 50 mL Teflon-lined autoclave, which was then heated at 120°C for 24 hours. After the autoclave cooled down to ambient temperature, we centrifuged the as-prepared UiO-66 at 6000 rpm to isolate it. We washed the white product three times with DMF and then three times with

MeOH. We subjected the washed samples to overnight drying under vacuum. NH_2-UiO-6 was prepared using the same method, substituting terephthalic acid with 2-aminoterephthalic acid (0.416 g).

Preparation of ZIF-8.

The room temperature method of ZIF-8 was conducted according to literature.[5] Zinc nitrate hexahydrate $Zn(NO_3)_2 \cdot 6H_2O$ (1mmol, 0.29g) and 2-methylimidazole (8mmol, 0.66g) were added to separate beakers containing 15 mL of MeOH, and the solutions were kept under continuous stirring at 300 rpm until complete homogeneity. Afterward, the metal solution was added drop-wisely to the ligand solution, and the final solution was kept under vigorous stirring for 24 hours. After this, the ZIF-8 particles were separated by centrifugation at 600 rpm, washed with fresh MeOH, and finally dried under vacuum for overnight to yield the final white ZIF-8 powder.

Preparation of Polyether Sulfone (PES) solution.

PES pellets (Aldrich 200 g, MW 58,000 g/mol) were heated at 110 °C for 24 h to remove humidity. Then, 25 wt. % of PES solution was prepared by adding PES pellets (12.5 g, 0.21 mmol) to dimethyl sulfoxide (DMSO) (37.5 g, 479.96 mmol), and the solution was kept under stirring in a sand bath heated at 60 °C. From the prepared solution, PES (1g, 0.017 mmol) was added to a vial containing DMSO (1g, 12.79 mmol) to obtain a 12.5 wt.% solution. The vial was kept under stirring in a sand bath heated at 65 °C. Following this step, the prepared solution underwent ultrasonication to remove eventual air bubbles and to ensure the homogeneity of the solution.

Preparation of MOF-PES MMMs.

Different MOF-PES composites of MIL-53(Al), NH_2-MIL-53(Al), UiO-66, NH_2-UiO-66 and ZIF-8 were prepared. Different MOF polycrystalline powders were added to small vials (11.11% wt/wt based on the total mass mixture). In each vial, DMSO (1 g, 12.79 mmol) was added to 0.25 g of each

MOF and kept under mechanical stirring at RT. The suspension was left until homogeneity. Afterward, 25 wt. % PES stock solution (1 g, 0.017 mmol) was gradually added to the previously prepared MOF solution and was kept under stirring in a heated sand bath.

The membranes were cast using an Automatic Film Applicator. The casting rate was applied at 25 mm/s with a casting distance of 280 mm. A 250 µm thickness casting knife was used. Finally, the solutions were poured on a glass plate, and then the membranes were formed by a nonsolvent immersion precipitation separation method (NIPS). The casted membranes were immersed into a deionized water bath for 24 h and washed several times. Finally, the membranes were removed from the coagulation bath, stored on cleaned tissue paper, and left to dry at RT overnight. The membranes were stored for characterization.

Characterization Techniques.

Physical Techniques. Elemental analyses (C, H, and N) were performed at the microanalysis service of the Dipartimento di Chimica e Tecnologie Chimiche of the Università della Calabria (Italy). Fourier-Transformed Infrared spectroscopy (FT-IR) spectra were recorded on a Nicolet-6700 spectrophotometer as KBr pellets. The morphology and composition characteristics of MOFs were evaluated using a Scanning Electron Microscopy (SEM).

X-ray Powder Diffraction Measurements. Fresh polycrystalline samples of each MOF, pristine PES membrane and MOF-PES MMMs were deposited on a flat plate with a 5 cm diameter prior to being mounted on a Bruker D2 PHASER Diffraction System with Cu-Kα radiation (λ = 1.54056 Å). Five repeated measurements were collected at room temperature (2θ = 2–50) and merged in a single diffractogram.

Gas Sorption. The N_2 adsorption isotherms at 77 K were carried out on crystalline MOF samples with a BELSORP MINI X instrument. Samples were activated at 70 °C under reduced pressure (10–6 Torr) for 16 h prior to carry out the sorption measurements. The Brunauer-Emmett Teller (BET) surface areas were calculated from the N_2 adsorption isotherm.

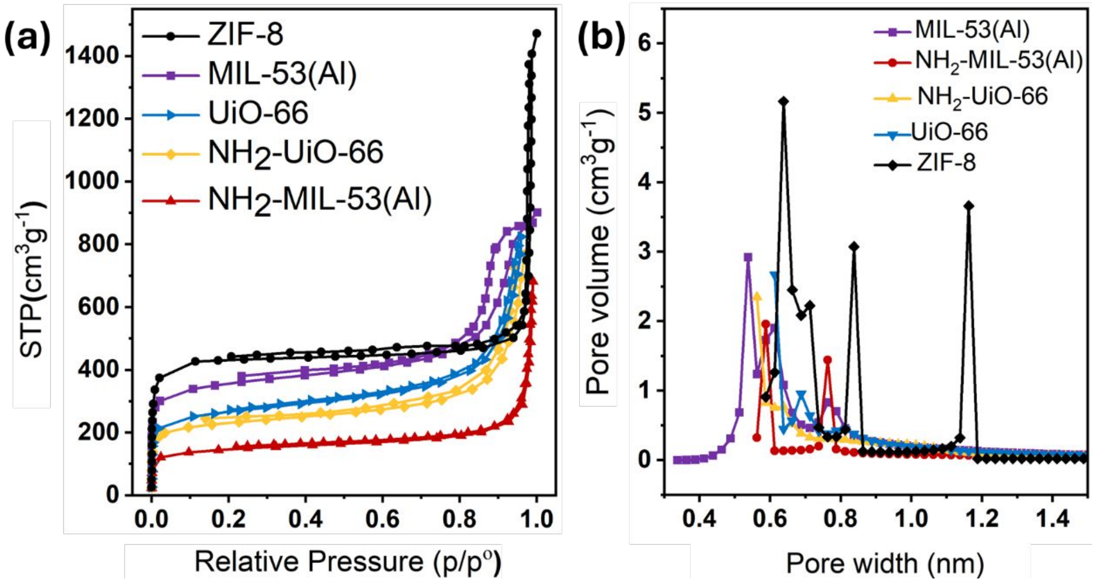

Figure S1: (a) N_2 adsorption isotherms (77 K) and **(b)** Pore size distribution of the activated MOFs.

FTIR spectra of MOFs and membranes.

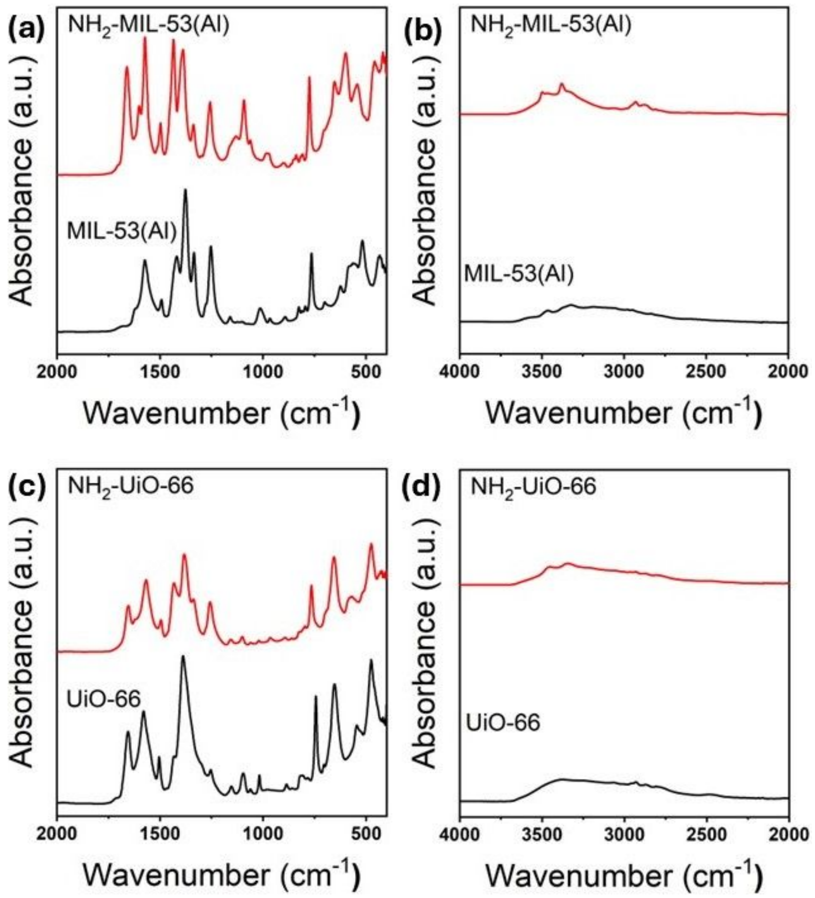

Figure S2: FT-IR spectra of (a and b) MIL-53(Al) (black) and NH_2-MIL-53(Al) (red), (c and d) UiO-66 (black) and NH_2- UiO-66 (red).

FTIR spectra of MOFs and NH_2-functionalized MOFs are shown in Figure S2. Figure S2a, and S2b shows the FTIR spectra of MIL-53(Al) and NH_2-MIL(53). The basic characteristics peaks in both MIL-53(Al) and NH_2- MIL-53(Al) appeared between 1400-1700 cm^{-1} which belong to symmetric and asymmetric stretching vibration of the carboxylic groups and between 1670-1690 cm^{-1} belong to uncoordinated ligand molecules entrapped within the pores.[6,7] Two characteristic peaks at 3380 and 3490 cm^{-1} in the FTIR spectra of NH_2-MIL-53(Al) are due to the symmetric and asymmetric vibrations of NH_2 functional group.[8] In addition, there is another new peak observed at 1230 cm^{-1} due to stretching vibration of the C-N bond. Figure 6c and 6d show the FTIR spectra of UiO-66 and NH_2-UiO-66. The peaks between 1400-1700 cm^{-1} are due to the stretching vibrations of C=C and C=O

bonds. Additional absorption bands are attributed to the C–H stretching modes of the aromatic and aliphatic parts of the ligand. Hydrogen-bonded water molecules physisorbed on the surface or in the inter-crystalline pores produce a strong and wide absorption band in the range of 3200-3600 cm^{-1}. Two distinct peaks at 3376 and 3457 cm^{-1} indicate the NH_2 group and the 2-aminoterephthalic acid of NH_2-UiO-66.

FTIR spectra of pristine PES membranes (Figure S3), shows the peak at 1578 cm^{-1}, 1485 cm^{-1} and 1407 cm^{-1} which are the stretching vibration of integral benzene rings.[50] The two peaks at 1321 cm^{-1} and 1295 cm^{-1} show the asymmetric stretching vibration, and the peaks at 1147 cm^{-1} represent the symmetric stretching vibration of the S=O bonds. [51] The stretching vibration at 1235 cm^{-1} corresponds to the C-O-O aromatic ether bonds of polyether sulfone.

For comparison the FTIR spectra of powder MOFs, PES membranes and MOF@PES MMMs are shown in the Figure S4. For MOF-PES MMMs, several new peaks appear at different wavenumbers besides the characteristic peaks related to the pristine MOFs and PES membranes, which indicate that the MOFs particles were successfully incorporated into the polymer matrix.

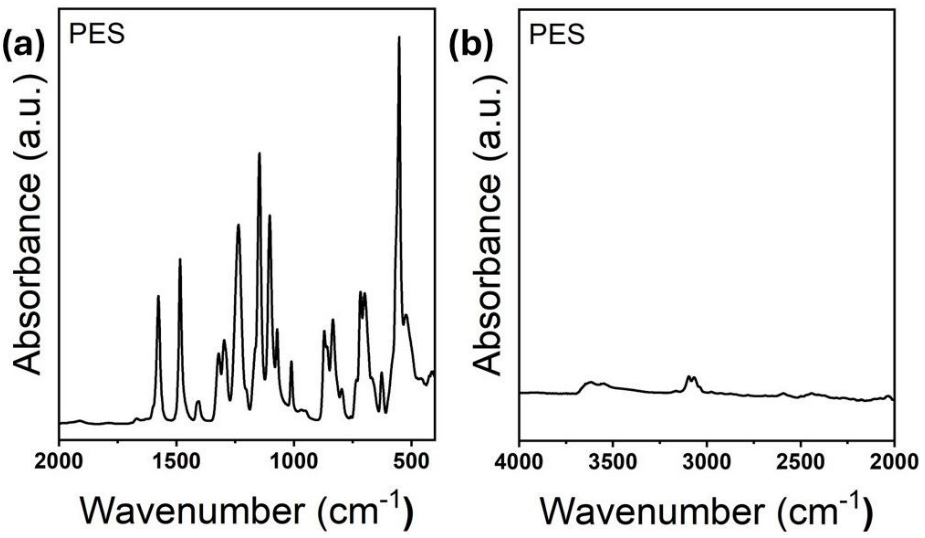

Figure S3: FT-IR spectra of PES membrane.

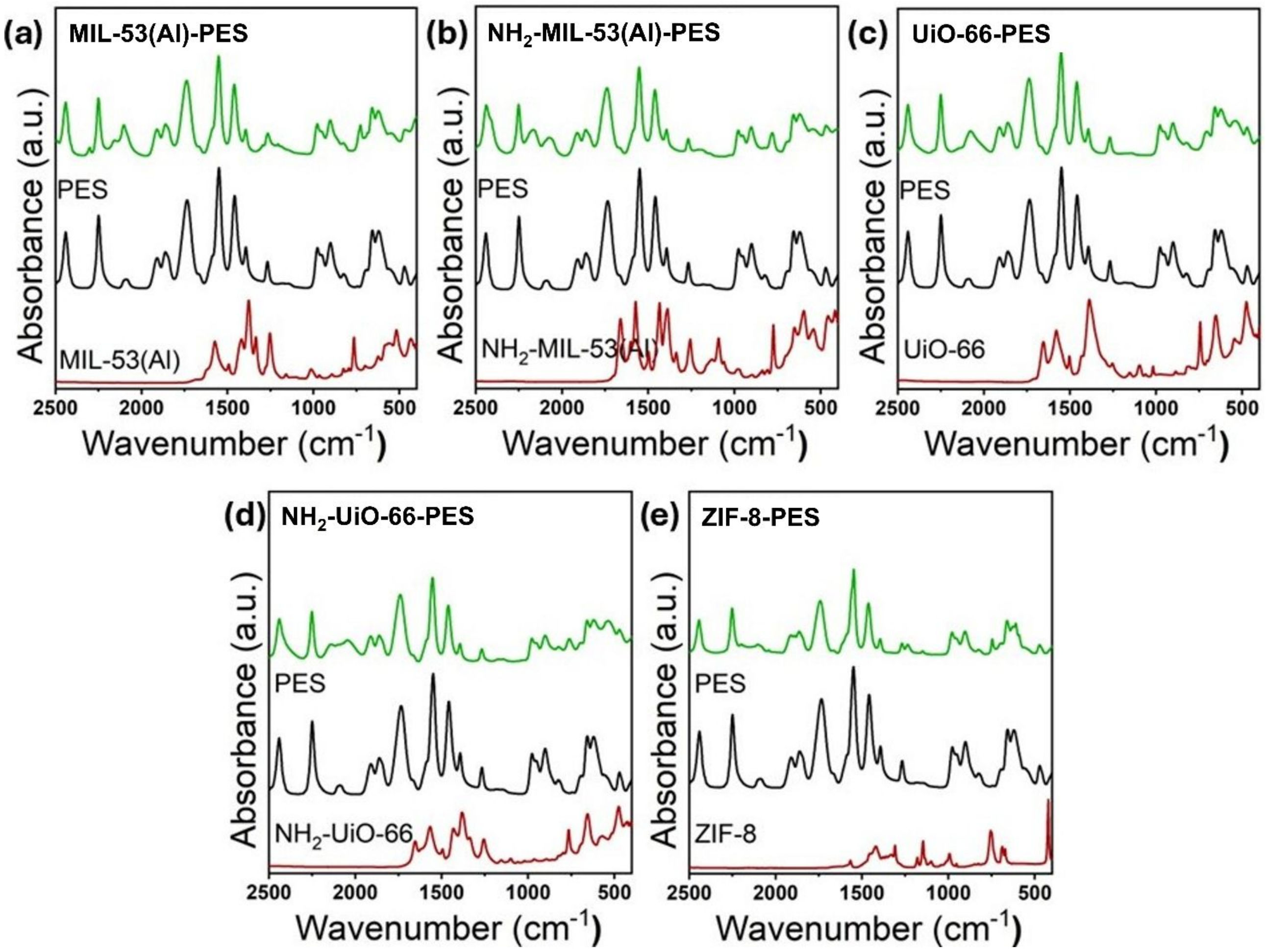

Figure S4: FT-IR spectra of powder MOFs (red), pristine PES (black) and MOF-PES (green). (a) MIL-53(Al), (b) NH_2-MIL-53(Al), (c) UiO-66, (d) NH_2- UiO-66, and (e) ZIF-8.

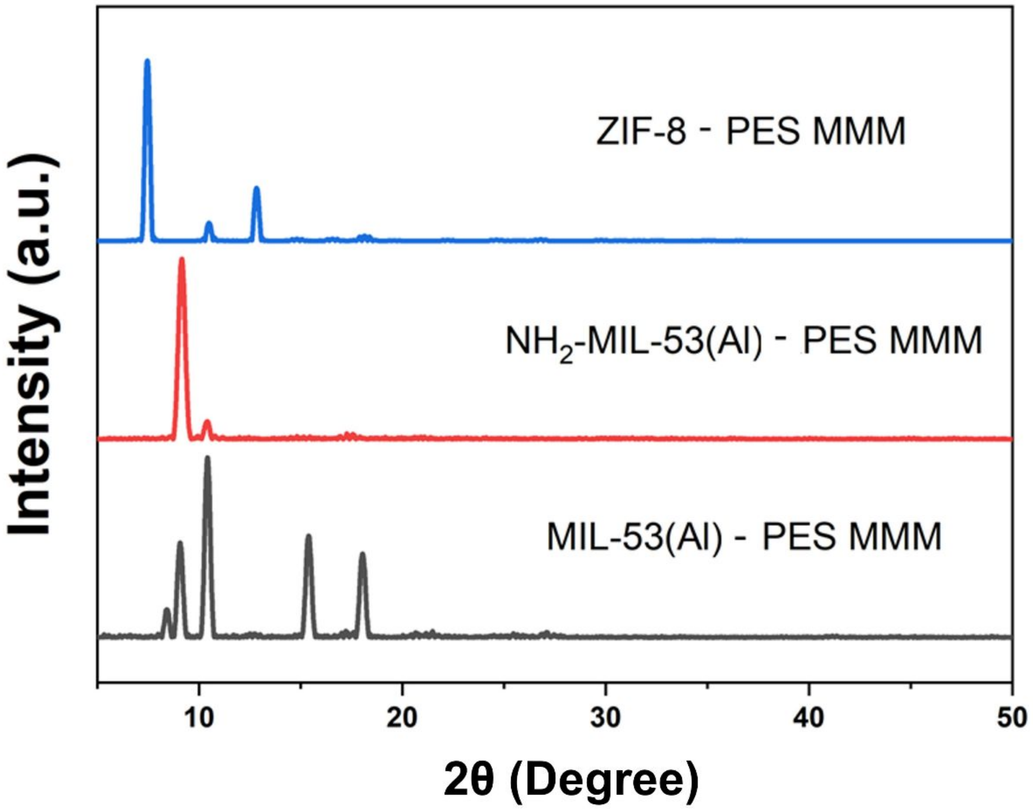

Figure S5. PXRD patterns of MIL-53(Al)-PES**,** NH_2-MIL-53(Al)-PES**,** and ZIF-8-PES MMMs after three adsorption–desorption regeneration cycles.

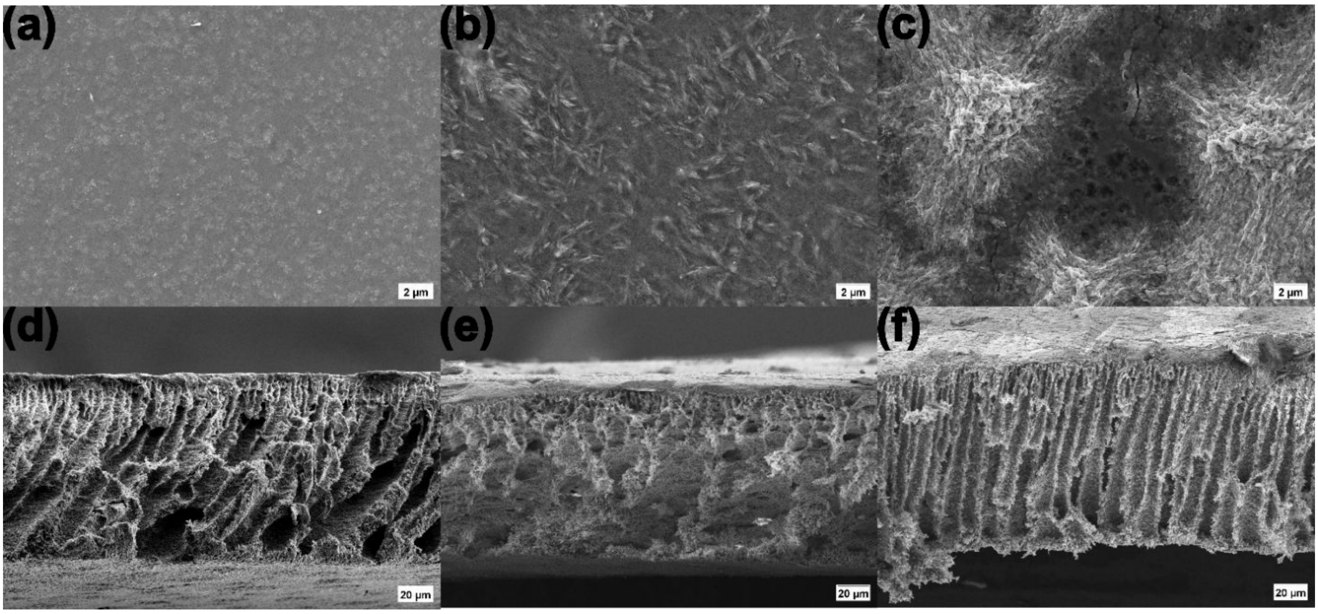

Figure S6. SEM micrographs of the (a–c) top surfaces and (d–f) cross-sections of MIL-53(Al)-PES**,** NH_2-MIL-53(Al)-PES, and ZIF-8-PES MMMs, respectively.

Table S1. Composition of oligo mineral water used for capture experiments (initial concentration of the interfering cations).

**Ions**	**Concentration (μg/L)**
Na^+	3369.2
K^+	1072.9
Mg^{2+}	929.7
Ca^{2+}	669.0

Table S2. Mean composition of Li-ion battery (smartphone battery).

**Li-ion battery metals**	**mg/g**
Li	38.4
Mn	63.5
Ni	70.0
Co	226.0

Table S3: Equilibrium maximum loading and removal efficiency for polycrystalline MOF samples soaked for 72 hours in 10 mL of an aqueous solution prepared with an oligomineral water containing common interfering ions (Table S1) and the target metal ions, at an initial concentrations of 200 mg/g (400 mg/L) for Ni(II) and Mn(II) and 600 mg/g (1200 mg/L) for Co(II), respectively. Capture experiments were conducted in triplicate and results are reported as average values ± 3 SD..

MOFs	Ni(II)		Mn(II)		Co(II)	
	mg/g	R(%)	mg/g	R(%)	mg/g	R(%)
**MIL-53(Al)**	19.4	9.3	10.8	5.4	160.3	26.6
**NH_2- MIL-53(Al)**	30.7	14.7	18.4	9.2	176.5	29.3
**UiO-66**	13.4	6.4	8.8	4.4	139.2	23.1
**NH_2-UiO-66**	19.4	9.3	16.2	8.1	153.0	25.4
**ZIF-8**	50.5	25.3	35.9	17.2	188.0	31.2

Table S4. Residual Ni^{2+} concentration[a] in an oligo-mineral aqueous solution (volume 100 mL) containing $Ni(NO_3)_2$ at an initial concentration of ca. 1 ppm, $Co(NO_3)_2$ at an initial concentration of ca. 3 ppm and $Mn(NO_3)_2$ at an initial concentration of ca. 1 ppm in presence of multi-ions as interfering media.[b]

Time (min)	**PES**	**MIL-53(Al)-PES**	**NH_2-MIL-53(Al)-PES**	**UiO-66-PES**	**NH_2-UiO-66-PES**	**ZIF-8-PES**
0	1.12	1.12	1.12	1.12	1.12	1.12
10	1.12	1.11	1.09	1.03	1.11	1.11
30	1.14	1.10	0.98	1.00	1.05	1.02
60	1.11	1.11	1.07	0.96	0.99	0.99
180	1.20	1.10	1.00	0.89	0.84	0.90
360	0.86	1.05	0.90	0.69	0.73	0.75
1440	0.97	0.62	0.45	0.58	0.50	0.34
2880	0.91	0.12	0.12	0.41	0.31	0.16
4320	0.86	0.03	0.03	0.29	0.20	0.07

[a]LOD: 0.015 ppb. [b]Each experiment was performed in triplicate and results are reported as average values ± 3 SD.

Table S5. Residual Co^{2+} concentration[a] in an oligo-mineral aqueous solution (volume 100 mL) containing $Ni(NO_3)_2$ at an initial concentration of ca. 1 ppm, $Co(NO_3)_2$ at an initial concentration of ca. 3 ppb and $Mn(NO_3)_2$ at an initial concentration of ca. 1 ppm in presence of multi-ions as interfering media.[b]

Time (min)	**PES**	**MIL-53(Al)-PES**	**NH_2-MIL-53(Al)-PES**	**UiO-66-PES**	**NH_2-UiO-66-PES**	**ZIF-8-PES**
0	3.22	3.22	3.22	3.22	3.22	3.22
10	3.16	3.19	3.05	2.79	3.11	3.04
30	3.22	3.24	2.71	2.67	2.96	2.81
60	3.10	3.18	2.94	2.56	2.81	2.69
180	3.17	3.16	2.84	2.26	2.45	2..45
360	2.43	3.08	2.59	1.87	2.14	205
1440	2.85	2.27	1.88	1.85	1.87	1.28
2880	2.68	0.81	0.61	1.59	1.16	0.61
4320	2.60	0.29	0.18	1.21	0.72	0.29

[a]LOD: 0.015 ppb. [b]Each experiment was performed in triplicate and results are reported as average values ± 3 SD.

Table S6. Residual Mn^{2+} concentration[a] in an oligo-mineral aqueous solution (volume 100 mL) containing $Ni(NO_3)_2$ at an initial concentration of ca. 1 ppm, $Co(NO_3)_2$ at an initial concentration of ca. 3 ppm and $Mn(NO_3)_2$ at an initial concentration of ca. 1 ppm in presence of multi-ions as interfering media.[b]

Time (min)	**PES**	**MIL-53(Al)-PES**	**NH_2-MIL-53(Al)-PES**	**UiO-66-PES**	**NH_2-UiO-66-PES**	**ZIF-8-PES**
0	0.99	0.99	0.99	0.99	0.99	0.99
10	0.98	0.88	0.92	0.68	0.95	0.97
30	0.97	0.91	0.81	0.62	0.92	0.90
60	0.98	0.90	0.87	0.55	0.89	0.84
180	0.96	0.92	0.82	0.50	0.80	0.74
360	0.74	0.93	0.38	0.38	0.71	0.62
1440	0.82	0.88	0.71	0.85	0.70	0.34
2880	0.73	0.74	0.54	0.72	0.51	0.15
4320	0.66	0.65	0.30	0.61	0.37	0.07

[a]LOD: 0.015 ppb. [b]Each experiment was performed in triplicate and results are reported as average values ± 3 SD.

Table S7. Removal efficiency (%) for Ni(II), Co(II) and Mn(II) of different MOF-PES membranes soaked in an oligo-mineral aqueous solution (volume 100 mL) containing $Ni(NO_3)_2$ at an initial concentration of ca. 1 ppm, $Co(NO_3)_2$ at an initial concentration of ca. 3 ppb and $Mn(NO_3)_2$ at an initial concentration of ca. 1 ppm in presence of multi-ions as interfering media. Capture experiments were conducted in triplicate.

	**Removal efficiency (R%)**		
	Ni(II)	Co(II)	Mn(II)
**PES**	23.4	19.0	33.0
**MIL-53(Al)-PES**	97.4	91.0	33.9
**NH2-MIL-53(Al)-PES**	97.4	94.3	69.9
**UiO-66-PES**	74.1	62.5	38.1
**NH_2-UiO-66-PES**	72.2	77.6	63.1
**ZIF-8-PES**	93.2	91.1	93.0

Table S8: Reusability of MOF-PES MMMs up to three adsorption–desorption cycles in term of Removal Efficiency (%).

MOF-PES MMMs	R% I Cycle			R% II Cycle			R% III Cycle		
	Ni(II)	Mn(II)	Co(II)	Ni(II)	Mn(II)	Co(II)	Ni(II)	Mn(II)	Co(II)
**MIL-53(Al)-PES**	97.4	33.9	91.0	96.6	34.5	91.45	96.8	33.5	90.9
**NH_2-MIL-53(Al)-PES**	97.4	69.9	94.3	98.3	70.3	94.12	97.6	70.5	93.3
**ZIF-8-PES**	93.2	93.0	91.1	94.5	92.5	90.57	93.4	93.2	90.8

Table S9: Metal Recovery (%) of MOF-PES MMMs up to three adsorption–desorption cycles.

MOF@PES MMMs	MR% I Cycle			MR% II Cycle			MR% III Cycle		
	Ni(II)	Co(II)	Mn(II)	Ni(II)	Co(II)	Mn(II)	Ni(II)	Co(II)	Mn(II)
**MIL-53(Al)-PES**	92.5	86.4	24.7	92.2	86.7	25.0	92.3	86.5	24.9
**NH_2-MIL-53(Al)-PES**	92.9	89.7	64.7	93.1	89.9	64.7	92.9	90.0	64.4
**ZIF-8-PES**	88.1	85.6	87.8	88.9	85.5	87.6	88.4	85.1	87.3

References.

1) Loiseau, T.; Serre, C.; Huguenard, C.; Fink, G.; Taulelle, F.; Henry, M.; Bataille, T.; Férey, G. A Rationale for the Large Breathing of the Porous Aluminum Terephthalate (MIL-53) Upon Hydration. *Chemistry - A European Journal* **2004**, *10* (6), 1373–1382. https://doi.org/10.1002/chem.200305413.

2) Molavi, H.; Hakimian, A.; Shojaei, A.; Raeiszadeh, M. Selective Dye Adsorption by Highly Water Stable Metal-Organic Framework: Long Term Stability Analysis in Aqueous Media. *Appl Surf Sci* **2018**, *445*, 424–436. https://doi.org/10.1016/j.apsusc.2018.03.189.

3) Molavi, H.; Zamani, M.; Aghajanzadeh, M.; Kheiri Manjili, H.; Danafar, H.; Shojaei, A. Evaluation of UiO-66 Metal Organic Framework as an Effective Sorbent for Curcumin's Overdose. *Appl Organomet Chem* **2018**, *32* (4). https://doi.org/10.1002/aoc.4221.

4) Aghajanzadeh, M.; Zamani, M.; Molavi, H.; Khieri Manjili, H.; Danafar, H.; Shojaei, A. Preparation of Metal–Organic Frameworks UiO-66 for Adsorptive Removal of Methotrexate from Aqueous Solution. *J Inorg Organomet Polym Mater* **2018**, *28* (1), 177–186. https://doi.org/10.1007/s10904-017-0709-3.

5) Zhou, K.; Mousavi, B.; Luo, Z.; Phatanasri, S.; Chaemchuen, S.; Verpoort, F. Characterization and Properties of Zn/Co Zeolitic Imidazolate Frameworks vs. ZIF-8 and ZIF-67. *J Mater Chem A Mater* **2017**, *5* (3), 952–957. https://doi.org/10.1039/C6TA07860E.

6) Feijani, E. A.; Mahdavi, H.; Tavasoli, A. Poly(Vinylidene Fluoride) Based Mixed Matrix Membranes Comprising Metal Organic Frameworks for Gas Separation Applications. *Chemical Engineering Research and Design* **2015**, *96*, 87–102. https://doi.org/10.1016/j.cherd.2015.02.009.

7) Rallapalli, P.; Prasanth, K. P.; Patil, D.; Somani, R. S.; Jasra, R. V.; Bajaj, H. C. Sorption Studies of CO2, CH4, N2, CO, O2 and Ar on Nanoporous Aluminum Terephthalate [MIL-53(Al)]. *Journal of Porous Materials* **2011**, *18* (2), 205–210. https://doi.org/10.1007/s10934-010-9371-7.

8) Chen, X. Y.; Vinh-Thang, H.; Rodrigue, D.; Kaliaguine, S. Amine-Functionalized MIL-53 Metal-Organic Framework in Polyimide Mixed Matrix Membranes for CO 2/CH 4 Separation. *Ind Eng Chem Res* **2012**, *51* (19), 6895–6906. https://doi.org/10.1021/ie3004336.

9) *Infrared and Raman Spectroscopy*; Schrader Bernhard, Ed.; Wiley, 1995. https://doi.org/10.1002/9783527615438.

10) Luo, M. L.; Tang, W.; Zhao, J. Q.; Pu, C. S. Hydrophilic Modification of Poly(Ether Sulfone) Used TiO2 Nanoparticles by a Sol-Gel Process. *J Mater Process Technol* **2006**, *172* (3), 431–436. https://doi.org/10.1016/j.jmatprotec.2005.11.004.
